# Resilient and Flexible Electrohydrodynamics Pumps for Human–Machine Interfaces

**DOI:** 10.1002/advs.202416502

**Published:** 2025-04-16

**Authors:** Yu Kuwajima, Amr Marzuq, Soraya Segawa, Yuya Yamaguchi, Yuhei Yamada, Takafumi Morita, Katrene Morozov, Haruto Iwasaki, Sota Suzuki, Hiroyuki Nabae, Vito Cacucciolo, Naoki Hosoya, Yasuaki Kakehi, Shingo Maeda

**Affiliations:** ^1^ Department of Mechanical Engineering Institute of Science Tokyo 2‐12‐1, Ookayama Meguro‐ku Tokyo 152‐8550 Japan; ^2^ Department of Mechanics, Mathematics and Management (DMMM) Politecnico di Bari Via Orabona 4 70125 Bari Italy; ^3^ Department of Engineering Science and Mechanics Shibaura Institute of Technology 3‐7‐5, Toyosu Koto‐ku Tokyo 135‐8548 Japan; ^4^ Research Center for Autonomous Systems Materialogy (ASMat) Institute of Innovative Research, Institute of Science Tokyo 4259, Nagatsuta‐Cho, Midori‐Ku Yokohama Kanagawa 226‐8501 Japan; ^5^ The University of Tokyo 7‐3‐1, Hongo Bunkyo‐ku Tokyo 113‐8654 Japan; ^6^ Department of Physics University of California Santa Barbara, Broida Hall Santa Barbara CA 93106 USA

**Keywords:** display, electrohydrodynamics, flexible, pump, prosthetic hand, resilient, soft actuator

## Abstract

Soft fluidic systems can be a versatile tool to design human–machine interfaces such as hydraulic actuators, liquid displays, and thermal haptics. Yet the bulkiness, noise, and rigidity of pumps and valves required for fluid circulation prevent their use in flexible and portable devices. This study introduces an electrohydrodynamic (EHD) driven flexible pump with resilience against dielectric breakdown. Previous EHD pumps, despite their excellent features such as quietness and high power density, suffer from dielectric breakdowns and subsequent permanent failures. This pump with novel electrode construction has the passive resilience to recover the insulation essential for EHD without any external input in the event of dielectric breakdown. The passive resilience of our pump is demonstrated in various scenarios. Notably, this pump withstands 100 dielectric breakdowns and maintains 90% of its performance. An active resilient system is also configured to enable continuous pumping. This system automatically removes bubbles and other impurities to recover flow generation. This pumps drive various soft fluid‐driven human‐machine interfaces like soft actuators, prosthetic hands, and tube‐format displays. The combination of passive resilience inherent in the pump and active resilience configured by the system ensures adaptability and robustness, setting the stage for the next generation of human–machine interfaces.

## Graphical abstract

This study introduces resilient and flexible electrohydrodynamic (EHD) pumps designed to enhance human–machine interfaces. These pumps are both quiet and compact, featuring a unique electrode configuration that overcomes the typical limitations associated with dielectric breakdowns and subsequent permanent failures. Remarkably, the pumps retain functionality even after enduring over 100 dielectric breakdowns, highlighting their robustness and reliability in various applications.

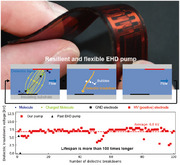

## 1 Introduction

The advancement of soft and resilient devices is crucial for the next generation of human‐machine interfaces. Utilizing soft materials in devices enhances safety for biological systems and provides adaptability to diverse environments, thereby facilitating coexistence with humans and other biological systems. However, the application of soft devices often entails unpredictable and uncontrollable environments, where the devices may be damaged. Therefore, just as the human body can heal itself after an injury, soft devices also need “resilience,” which entails the incorporation of mechanisms that enable engineered recovery within the system. Recently, the concept of resilience has begun to be integrated into soft electronics,^[^ ^1^, ^2^ ^]^ sensors,^[^ ^3^, ^4^ ^]^ and actuators.^[^ ^5^, ^6^, ^7^, ^8^, ^9^, ^10^ ^]^

Fluid actuation is a fundamental technology in soft devices.^[^ ^11^, ^12^, ^13^ ^]^ Soft fluidic devices enable numerous applications, including artificial muscles, grippers, haptics, thermal transport, and displays. In fluidic devices, pumps play an important role in generating pressure and transporting fluid, which directly affects the overall performance of the fluidic device. Traditional mechanical pumps, made up of rigid parts like impellers and gears, are bulky and generate noise, which is undesirable for human–machine interfaces. In recent years, more and more soft pumps composed of flexible materials have been proposed.^[^ ^14^, ^15^, ^16^, ^17^, ^18^, ^19^, ^20^, ^21^ ^]^ However, few existing soft pumps have both sufficient power and resilience.

Electrohydrodynamic (EHD) pumps are gaining attention as an alternative to mechanical pumps due to their advantages such as quiet operation, compact size, lightweight, and low heat generation.^[^ ^22^, ^23^, ^24^, ^25^, ^26^, ^27^, ^28^, ^29^, ^30^ ^]^ In EHD pumps, an electric charge is injected from an electrode into a dielectric liquid, which moves according to an electric field to produce flow.^[^ ^31^ ^]^ No need for mechanical parts has spurred the proposal of soft EHD pumps.^[^ ^7^, ^32^, ^33^, ^34^, ^35^ ^]^

Irreversible failures caused by dielectric breakdown are a common issue in EHD pumps, which require high electric fields on the order of kV/mm for operation. For instance, EHD pumps with comb‐shaped electrodes patterned on an insulating substrate can lead to the breakdown of the substrate itself, as the electric field is strongest on the substrate's surface between the electrodes. Dielectric breakdown occurs stochastically with the probability that depends on the strength of the electric field.^[^ ^36^ ^]^ Weaken the electric field reduces the likelihood of breakdown, but this also lowers the pump's output in terms of pressure and flow rate. In EHD pumps, enlarging the electrode area increases the pumping performance, but also raises the risk of dielectric breakdown.^[^ ^37^ ^]^ Environmental factors, liquid degradation, and contaminants can also cause dielectric breakdown.^[^ ^38^ ^]^ These challenges hinder long‐term operation, scaling of pumping, and use in diverse environmental conditions. The key to advancing the next generation of EHD pumps lies in integrating resilience, ensuring they can maintain pumping even after experiencing dielectric breakdowns. This capability will extend the lifespan of the pumps and the scalability of their pumping. In addition, this attempt can ease the limitations of the working liquid in EHD pumps and allow new applications.

**Figure 1:**
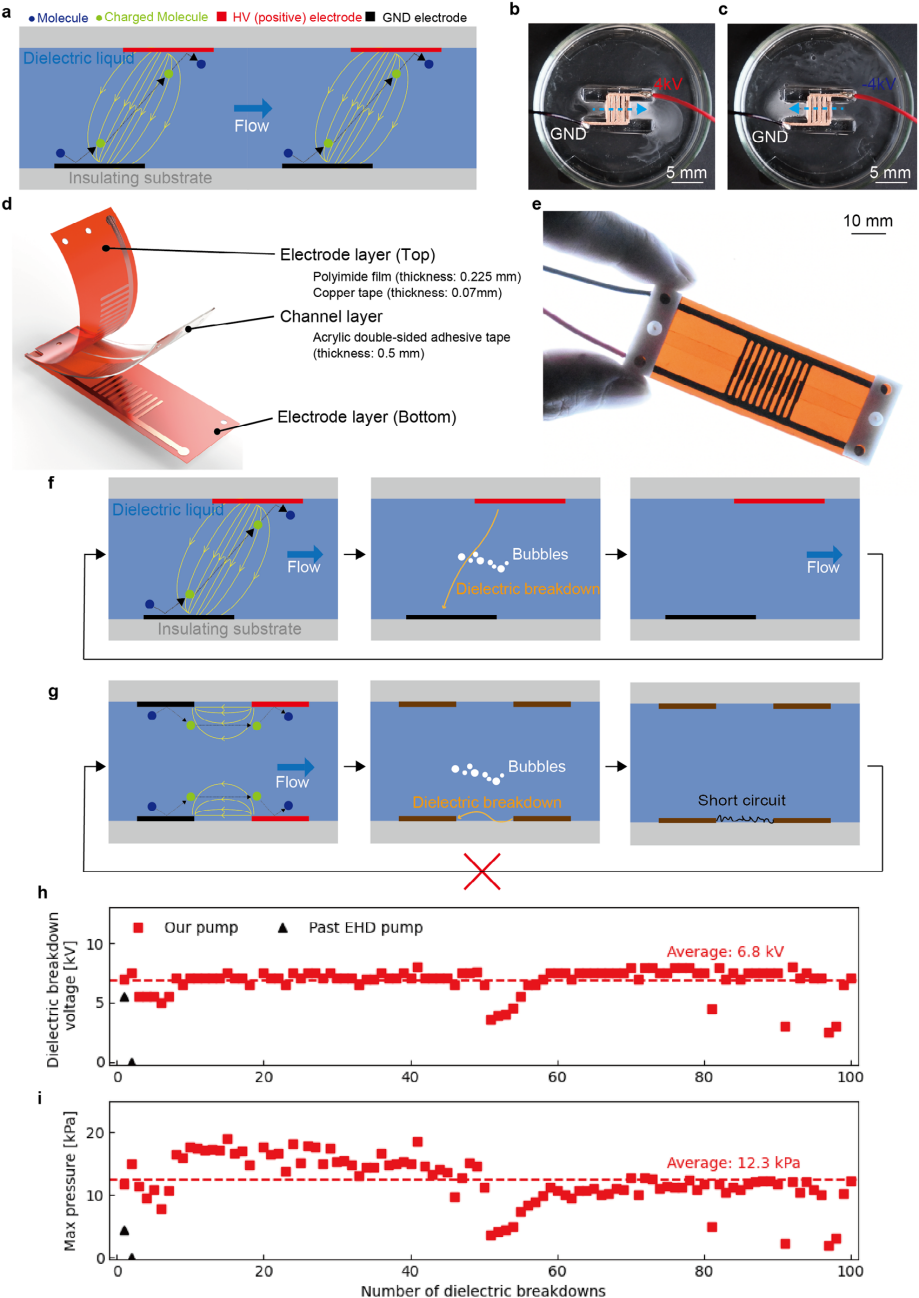
Flexible EHD pumps with passive resilience to dielectric breakdown. a) Mechanism of EHD. b,c) Visualization of bidirectional flow by a novel electrode structure. d) The pump comprises three flexible layers: upper and lower layers with comb‐shaped copper electrodes on a polyimide film, and a middle layer with flow channels cut from a double‐sided acrylic adhesive sheet. e) The developed pump. f) The structure of our pump limits the path of dielectric breakdown to dielectric liquid only. Even if the dielectric breakdown occurs, only bubbles are generated and the insulation of the insulating substrate is maintained without active mechanism. The pump will work again once its inside is filled with dielectric liquid. g) The failure mechanism due to the dielectric breakdown of the comb‐shaped electrode EHD pump. h,i) The dielectric breakdown voltage and maximum pressure of our pump and a past EHD pump are compared.

Here we present resilient and flexible EHD pumps. This pump is designed to be passively resilient, which means it inherently resists failures caused by dielectric breakdown without any active mechanisms. This pump is arranged so that the high voltage (HV) and GND comb electrodes on different insulating substrates (**Figure** **1**a,d). This limits the path of dielectric breakdown to the dielectric liquid and prevents the dielectric breakdown of the insulating substrate (Figure 1f). This strategic placement ensures that, even in the event of a dielectric breakdown, the insulation necessary for EHD pumping is maintained. We have demonstrated that our pumps are passively resilient in various situations that lead to dielectric breakdown, including environments contaminated with conductive liquids, bubbles, and the application of HV. In repeated breakdown tests with HV applied, our pump withstood 100 breakdowns with an average breakdown voltage of 6.8kV and an average pressure of 12kPa (Figure 1h,i). 90% of the dielectric breakdown voltage and pressure were maintained compared to the performance until the first dielectric breakdown. Also, this performance is noteworthy compared to past EHD pumps with comb electrodes for HV and GND on the same substrate, which do not work after the first dielectric breakdown. In addition, the newly adopted electrode design of the EHD pump can be fabricated using digital equipment and be easily expanded. Our pumps generated a maximum pressure of 48 kPa and a maximum flow rate of 63 mL min^−1^.

To ensure continuous operation following dielectric breakdowns, our pumps must remove any impurities, such as bubbles–whether introduced externally or generated by dielectric breakdown–and fully replenish the dielectric fluid within the system. Building upon the inherent passive resilience of our pumps, we have developed an active resilient system. This system consists of two pumps arranged in series. When a dielectric breakdown occurs in one pump, the other automatically activates to remove any impurities, ensuring that the system continues to operate uninterrupted. This configuration significantly enhances the reliability of the application by ensuring sustained operation.

Previous studies on EHD pumps have not focused on addressing dielectric breakdowns, despite their potential to cause catastrophic failures. Simply limiting the applied voltage, while increasing the safety factor, may prevent dielectric breakdowns to some extent. However, this approach leaves the system vulnerable to external disturbances and fails to fully exploit the potential performance of the pumps. In our research, we have developed a new type of EHD pumps that incorporate both active and passive resilience. Our pump is designed to allow dielectric breakdowns, thereby maximizing performance in unpredictable everyday environments. This innovative strategy highlights the versatility and extensive utility of our pumps in various applications, such as pouch actuators, McKibben artificial muscle‐driven prosthetic hands, and tube‐format displays. Our pumps meet the technical requirements of adaptability, durability, portability, and quiet operation for the next generation of human‐machine interfaces. They are ideally suited to enhance the interaction between humans and machines.

## 2 Results and Discussion

### 2.1 Mechanism, Design, and Fabrications

Our pump operates based on the EHD mechanism when an electric field is applied between its electrodes(Figure 1a). Under a high electric field, electric charges are injected from the electrodes into the dielectric liquid. The electric charges are accelerated by the electric field, transferring momentum to neutral liquid molecules along the way. In the structure of this pump with electrodes of different polarity on the top and bottom surfaces, the path of dielectric breakdown is presumed to occur in the dielectric liquid with a high electric field. Therefore, even if a dielectric breakdown occurs, only bubbles are generated in the dielectric liquid, the insulation is restored again, and pumping can be achieved by re‐applying voltage (Figure 1f).

Various electrode shapes have been proposed to enhance their pumping performance. Among them, comb‐type electrodes can be fabricated with versatile technologies such as plotting cutter,^[^ ^32^ ^]^ screen printing,^[^ ^33^ ^]^ and vapor deposition,^[^ ^39^ ^]^ and the output can be easily expanded since manual electrode placement is not required. Furthermore, EHD pumps with comb‐shaped electrodes can change the direction of flow by switching the polarity of the voltage to the electrodes thanks to their symmetrical structure. Despite these excellent features, EHD pumps with comb‐shaped electrodes are vulnerable to dielectric breakdown and prone to permanent failure(Figure 1g). Since the positive and negative electrodes are formed on the same insulating substrate, the surface of the insulating substrate has the highest electric field when voltage is applied. Therefore, dielectric breakdown tends to occur on the surface of the insulating substrate, causing thermal damage to the insulating substrate and possibly resulting in permanent failure. The new comb electrode arrangement proposed in this study can withstand dielectric breakdown while having the extensibility and bi‐directionality of comb electrodes.

The direction of the electric field formed by the electrodes is important to generate net flow (Figure S1, Supporting Information). When the shift *a* of the GND‐HV electrodes is too small, the electric field in the channel direction will be small and no flow will be generated. When *a* is too large, the distance between the electrodes will increase and the electric field will be small. The shift *d* of the GND‐GND and HV‐HV electrodes is also an important parameter; when this value coincides with *a*, the flow is canceled out. *d* must be sufficiently larger than *a*, but the size of pump will increase. Here, the optimal values *a* = 0.5 and *d* = 2.1 were chosen from the theory and simulation of the electric field to maximize the fluid power per size (Figures S2–S4, Supporting Information). In theory, fluid power per size is derived from the forward electric field generated between electrodes and the backward electric field generated between adjacent electrode pairs (See Note S1, Supporting Information, for details). A prototype pump with four pairs of electrodes was developed using this design, and bi‐directional flow was observed to be generated by switching the polarity of voltages (Figure 1b,c; Movie S1, Supporting Information).

Figure 1d, e shows the structure and developed pump with 10 pairs of electrodes. The structure of our pump consists of three flexible layers: the top and bottom layers are copper electrodes patterned in a comb shape on a polyimide film, and the middle layer is a double‐sided adhesive acrylic material.

We fabricated pumps consisting of three layers by a digital fabrication method (Figure S5, Supporting Information). Electrode layers (top and bottom): First, copper tape is attached to polyimide film. Using a plotting cutter, the copper tape is patterned into a comb shape, and the outer frame is cut out. Unnecessary copper tape is manually removed. Channel layer (middle): Acrylic elastomer with double‐sided adhesive is cut into channel shapes using a laser cutting machine. These three layers were assembled using a jig (Figure S6, Supporting Information). The key is to arrange them to match the design values *a* and *d* (See Note S1 for details, Supporting Information).

### 2.2 Passive Resilience of Pumps

We compared the performances of our pump and the pump of comb‐shaped electrodes in repeated dielectric breakdown tests(Figure 1g,h). The evaluation was performed by means of an experimental setup (Figure S7, Supporting Information). After the first dielectric breakdown, the pump with the comb‐shaped electrodes showed a dramatic decrease in dielectric breakdown voltage and pressure. On the other hand, our pump maintained its dielectric breakdown voltage and pressure after 100 dielectric breakdowns.

The fundamental cause of permanent failures in previous EHD pumps was the formation of a permanent carbonized conductive path between the HV and GND electrodes on the insulating substrate during dielectric breakdown. Dielectric breakdown tends to occur where the electric field intensity is highest, namely the shortest path between the HV and GND electrodes. Therefore, in traditional comb electrode EHD pumps with both HV and GND electrodes on the same substrate, the insulating substrate becomes the path for dielectric breakdown. The significant current during a dielectric breakdown generates heat, which forms a permanent conductive path on the insulating substrate, electrically connecting the HV and GND electrodes. The inability to apply voltage after one breakdown and the observed blackening between the HV and GND electrodes (Figure S8b, Supporting Information) suggest that a conductive path had been formed between the HV and GND electrodes.

In our pump design, we positioned the HV and GND electrodes on separate insulating substrates to ensure that the maximum electric field strength occurs within the dielectric fluid. This design change restricts the breakdown path to within the working fluid, preventing the formation of a permanent conductive path. The fact that our pump was able to withstand 100 cycles of dielectric breakdown and apply high voltages confirms this hypothesis. Our observations after 100 breakdowns show blackening on the insulating substrate (Figure S9d, Supporting Information); however, this indicates the possibility of conductance between GND‐GND or HV‐HV electrodes, not between HV and GND.

Although the conductance between GND‐GND or HV‐HV electrodes could potentially alter the designed electric field and degrade pump performance, the ability to maintain high‐pressure levels throughout 100 repeated breakdowns suggests that the insulation between these electrodes was sufficiently maintained.

**Figure 2:**
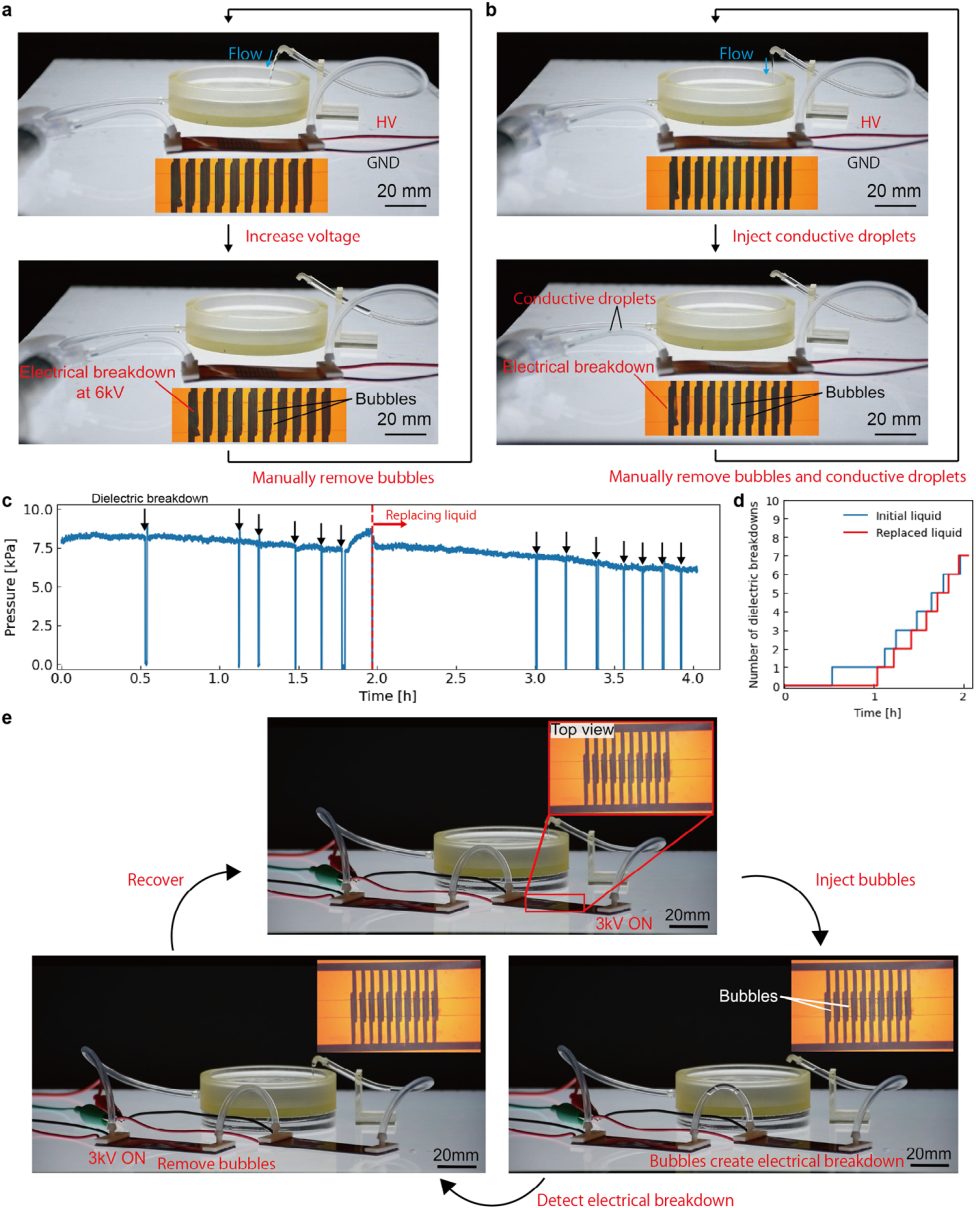
Passive resilience in various situations and active resilience system. In a fluid system with circulation by a pump, dielectric breakdowns occur due to a) increasing voltage and b) injection of a conductive liquid. After manually removing the bubbles generated by dielectric breakdown, the voltage is re‐applied and flow is generated. c) Long term pumping at 5kV voltage. The working liquid is replaced at approximately 2 h, and this action reduces the frequency of dielectric breakdown. d) Number of dielectric breakdowns versus time: The number of electrical breakdowns is low until 1 h, after that, the frequency begins to increase dramatically. e) Active resilient system by two pumps in series. The right side pump normally generates flow, and when a dielectric breakdown is detected, the left side pump automatically removes the bubbles that inhibit flow generation and recovers to a pumpable state.

We observed the behavior of the pump as we gradually increased the voltage applied to it (**Figure** **2**a; Movie S2, Supporting Information). As the voltage was increased, the pump produced a larger flow. At 6 kV, an electrical breakdown and a bubble appeared inside the pump. After manually removing the bubbles and re‐applying the voltage, the pump again produced flow. Dielectric breakdown occurred not only when the input voltage was increased, but also in situations where the system was contaminated with conductive liquids or bubbles, and when voltage was applied over a long duration. In each scenario, our pumps proved to be resilient. A voltage of 2 kV was applied to the pump and colored water was injected into the system. Even after the dielectric breakdown occurred, the pump generated the flow by re‐applying the voltage and replacing liquid (Figure 2b; Movie S3, Supporting Information). A voltage of 5 kV, which is about 75% of the average electrical breakdown voltage in the repeated dielectric breakdown test, was applied for 4 h (Figure 2c). Despite the constant voltage input, dielectric breakdowns were observed. A total of seven dielectric breakdowns were observed in the first 2 h, and the frequency of these dielectric breakdowns tended to increase with time (Figure 2d). We assume that applying voltage for the long term degrades the dielectric liquid and lowers the dielectric breakdown voltage. When the dielectric liquid was replaced after 2 h, the frequency of the dielectric breakdowns decreased significantly, and the frequency of the electrical breakdowns tended to increase with time. This finding is an important guideline for developing maintenance strategies during operation. We defined the safety factor as the ratio of the dielectric strength of the working fluid to the electric field strength applied during long‐term operation tests. Importantly (Table S2, Supporting Information), we have successfully demonstrated the pump's operation under a safety factor of 1.0 for a duration of 4 h. This demonstrates our pump's unique ability to operate and maximize output at voltages close to the theoretical dielectric breakdown threshold, representing a significant advancement over traditional designs that require larger safety margins.

### 2.3 Active Resilience of Multi‐pump System

Bubbles cause dielectric breakdown and obstruct flow. If bubbles are not removed, they will stack on the voltage‐applied electrodes. This interferes with the process of injecting charge into the dielectric liquid, which is necessary for EHD flow. Therefore, after dielectric breakdowns, it is necessary to remove the bubbles to obtain the net flow again. Here, we proposed an active resilient fluid system that automatically removes the bubbles, and generates a continuous flow (Figure 2e; Movie S4, Supporting Information). The fluid system employs two pumps connected in series. The voltage to each pump is controlled by the circuit developed. Normally, 3 kV is applied to the pump (right side) to generate flow. When a dielectric breakdown occurs, the current at that time is detected. That triggers the applying voltage to the pump (left side). The pump (left side) generates flow for a period of time to remove bubbles from the system. Then, the system recovers to a pumpable state. After that, voltage is again applied to the pump (right side) and the system generates flow. This system configuration is only an example of the possibilities, but it shows a solution to generate a continuous flow. In actual applications, this system configuration can be further customized and optimized to reduce downtime and maintenance.

### 2.4 Performance of Scaled‐up Pumps

Pressure and flow rates were improved by increasing the number of electrode pairs and by increasing the overlap and channel width of the electrodes. In previous studies, modularized EHD pumps were connected in series and parallel to extend pressure and flow rate.^[^ ^27^, ^33^ ^]^ While this has the advantage that only broken pumps can be replaced, it also results in a cumbersome system with extra tubing connections. Our pumps have a simple structure and resilience, so it is possible to expand the output of a single pump without modularization.

**Figure 3:**
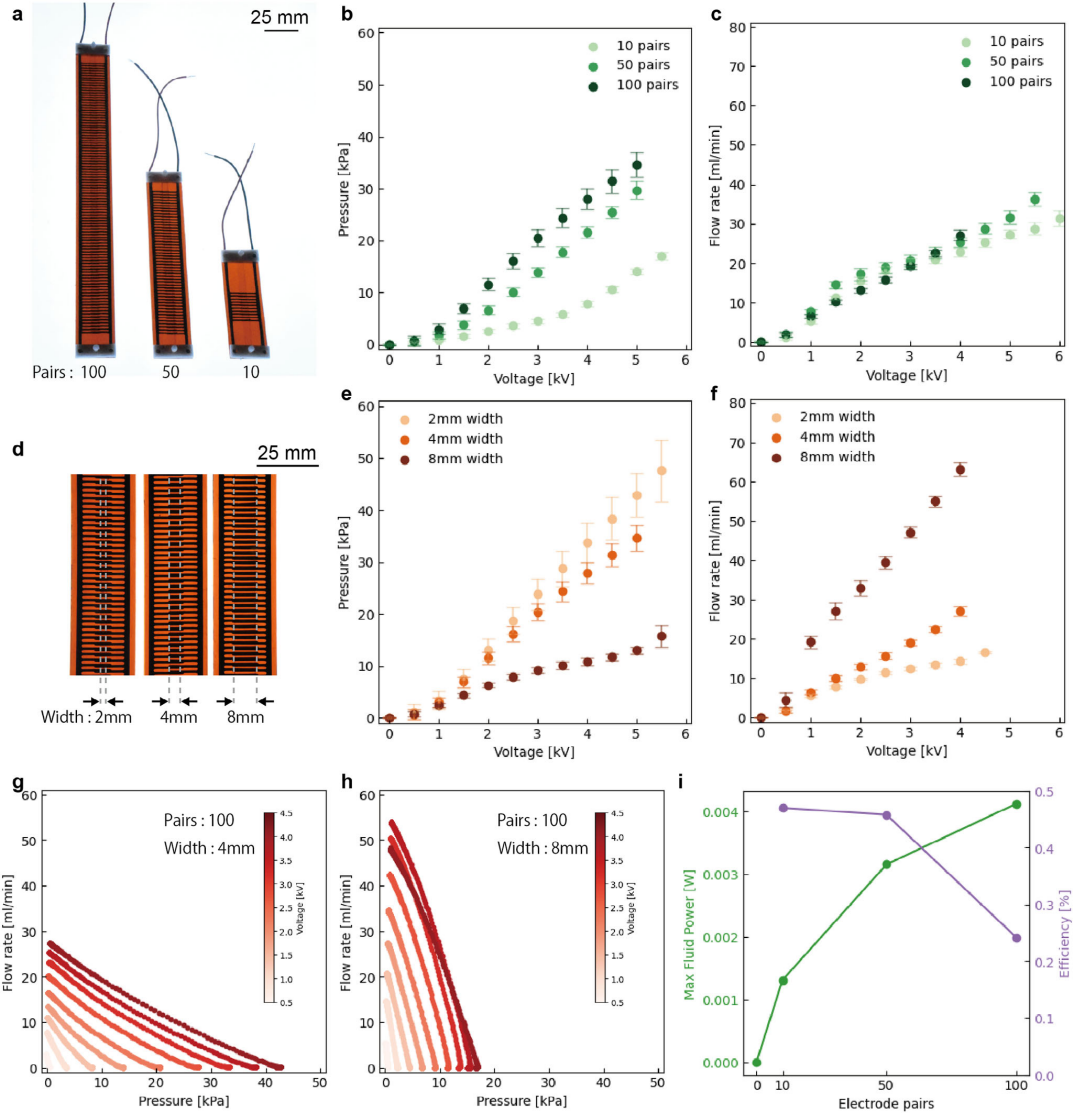
Performances of scaled‐up pumps. a) Pumps with 10, 50, and 100 electrode pairs with 4mm width and their b) pressure and c) flow rate performance. d) Pumps with 100 electrode pairs with 2, 4, 8mm width and their e) pressure and f) flow rate performance. g,h) The pressure‐flow rate curve of the pump with 100 electrode pairs of 4,8 mm width. i) Maximum fluid power and efficiency versus number of electrode pairs.

In this paper, we fabricated pumps with 10, 50, and 100 pairs of electrodes with channel widths of 4 mm (**Figure** **3**a) and pumps with 100 pairs of electrodes with channel widths of 2, 4, and 8 mm (Figure 3d). Table S1 (Supporting Information) shows the detailed values of the geometry. We achieved a maximum of 48 kPa with the pump at 2 mm channel width and a maximum of 63 mL min^−1^ at 8 mm channel width with 100 electrode pairs. As the number of electrodes increases, the pressure increases while the flow rate remains the same (Figure 3b,c). The pressure decreased as the flow path width increased, while the flow rate tended to increase (Figure 3e,f).

To investigate further, we also evaluated the pump with 100 pairs of electrodes with 16 mm channel width. Although the pump with a wider channel acts EHD on more dielectric liquid and higher flow rates can be expected, the flow rate was less than the pump with 4 mm width. During the experiment, we observed deformation of the electrode layers on the top and bottom surfaces of the flow channel in a pump with a channel width of 16 mm. We presume that this deformation, i.e., the increased distance between the electrodes, has led to a decrease in the electric field and pressure and a limit to the expansion of the flow rate. The pressure and flow rate of an EHD pump increases proportionally to the square of the electric field based on standard theory,^[^ ^31^ ^]^ however, actual characterization often does not follow this theory.^[^ ^35^, ^40^, ^41^ ^]^ Our pumps also show a different trend from the theory above a certain threshold and become proportional to voltage (Figure S10, S11, Supporting Information). This divergence, also noted in previous EHD pump studies, has not yet been resolved within the community. One contributing factor may be high variability because of the manual fabrication process. In the case of our pumps in particular, there is also the possibility that vortices will be generated due to deformation of the flow path. In future research, we plan to implement more stringent manufacturing protocols and investigate designs to minimize the variability and deformation of the flow path.

The pressure‐flow rate characteristics were evaluated for pumps with 100 electrode pairs and flow channel widths of 4 and 8 mm (Figure 3g,h; Figure S12, Supporting Information). For both pumps, the relationship between pressure and flow rate is linear, a trend that has been confirmed in previous soft EHD pumps.^[^ ^35^ ^]^ As the number of electrode pairs increases, the maximum fluid power, the product of pressure and flow rate, increases (Figure 3i). On the other hand, the efficiency at maximum fluid power tends to decrease as the number of electrode pairs increases. As shown in Figure 3b,c, the flow rate does not depend on the number of pairs, and the pressure increases with the number of pairs and saturates, so the maximum fluid power also increases and shows a tendency to saturate. The efficiency should be constant for a given number of pairs, but our pumps show a decrease in efficiency because the pressure is not linearly related to the number of pairs. At 100 pairs, the maximum fluid power and efficiency were 4.12 mW and 0.24%, respectively.

Our pumps are resilient, yet have standard performance compared to previous soft EHD pumps (Figure S13a, Supporting Information). Furthermore, the performance per pump size is equivalent to that of commercially available miniature pumps (Figure S13b, Supporting Information). Significantly, our pumps surpass all other performance metrics except for specific pressure compared to the comb electrode EHD pump. In our pump, the outer extent of the flow path has little effect on performance. Therefore, optimization of the design is expected to further improve the performance per size.

**Figure 4:**
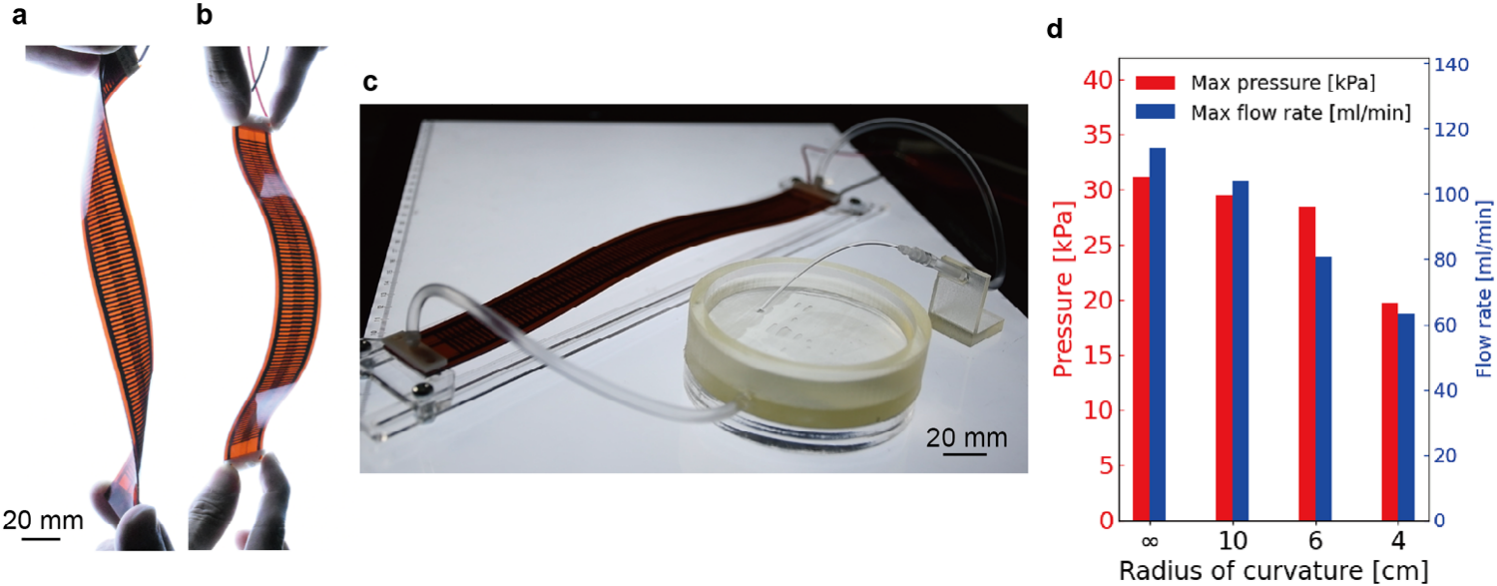
Flexibility of pumps. The pump itself can be a) twisted and b) bent. c) Demonstration of a jet stream in a bent state by the pump with 100 pairs and 8 mm width. d Pump (100 pairs, 4 mm width) performance versus bending curvature at 4 kV.

One of the attractive features of our pumps is the compliance obtained from their flexible materials. Our pumps tolerate external forces such as torsion and bending (**Figure** **4**a,b), and can accommodate unexpected inputs expected in everyday life. This flexibility also allows for installation on curved surfaces and efficient use of limited space, such as the human body. As Figure 4c and Movie S5, Supporting Information, show, the pump (100 electrode pairs, 8 mm electrode width) produces a jet flow at bending status. We evaluated the performance of the pump (100 electrode pairs, 4 mm channel width) in the bending state at 4 kV applied (Figure 4d). Both maximum pressure and flow rate tended to decrease at smaller radii of curvature. This decrease in performance is thought to be due to changes in the shape of the electric field and fluid‐specific pressure drop caused by bending. At a radius of curvature of 4 cm, pressure and flow decreased by 36% and 45%, respectively, compared to the unbent condition. And dielectric breakdown is more likely to occur when the pump is in a bending condition. This is thought to be due to the decrease in distance between electrodes caused by bending. The resilience of our pump to dielectric breakdown overcomes this problem as well.

### 2.5 Soft Fluidic Applications

Resilient, quiet, flexible, portable, and with high fluid output, our pumps enable soft fluid system‐based human–machine interfaces. In uncontrolled environments, such as everyday life, the risk of dielectric breakdown increases due to exposure to sudden shocks and external stresses. The resilience of our pumps ensures that users can use them for the long term with peace of mind and provides great value in the user experience. Here we demonstrated our pumps with a pouch actuator that can be customized for the application, a prosthetic hand with McKibben artificial muscles with properties similar to natural skeletal muscle, and a tube format display with colored water.

**Figure 5:**
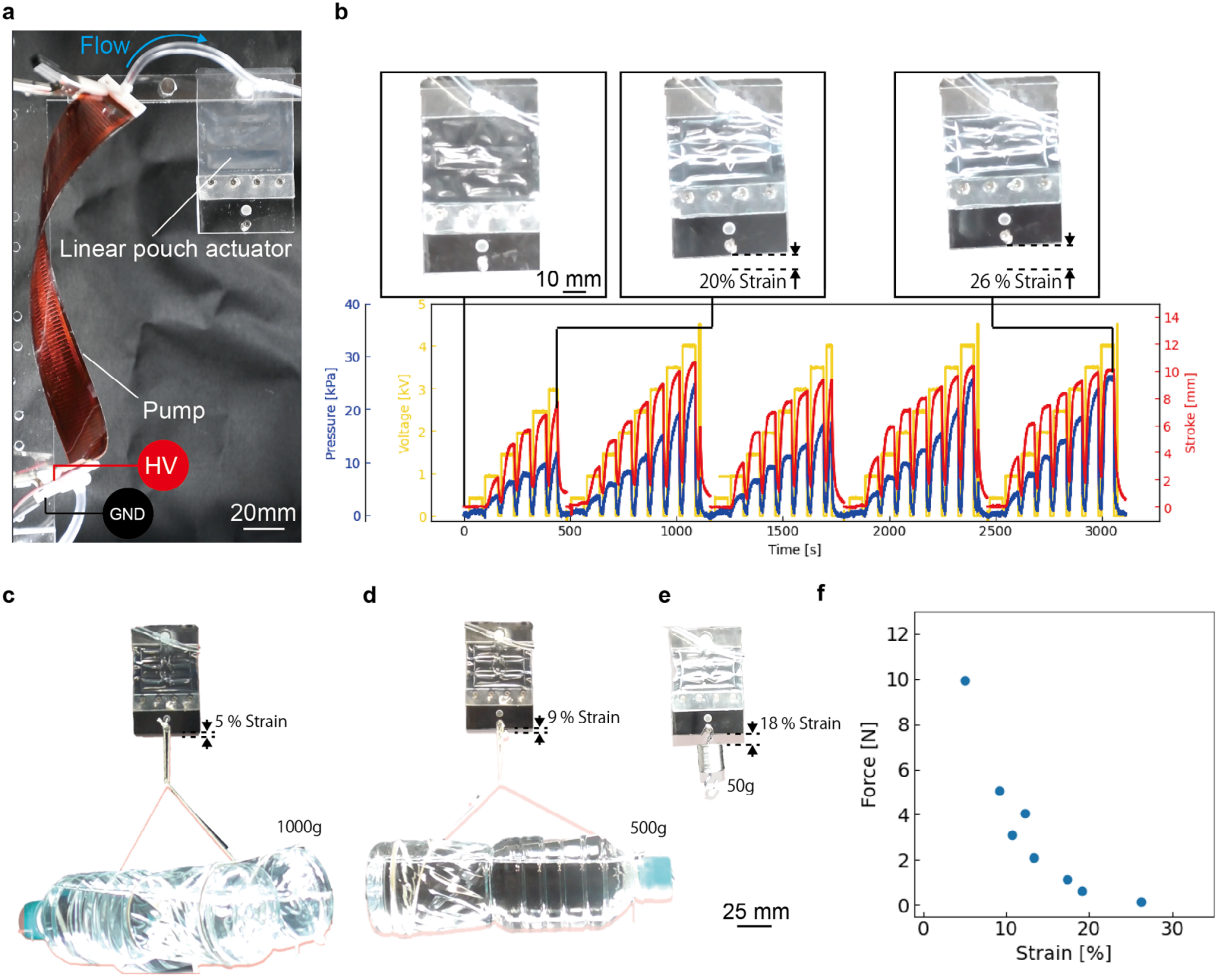
A linear pouch actuator driven by a twisted pump. a) System configuration. The twisted pump (100 pairs,4 mm width) and a linear pouch actuator are connected via tubing. b) Resilient actuation. Strokes are generated while experiencing a total of five dielectric breakdowns. Strains of a linear pouch actuator at c) 1000, d) 500, and e) 50 g load. f) Relationship between strain and force.

Pouch actuators are gaining attention as an option for soft actuators thanks to their simple structure, thinness, lightness, flexibility, high power density, and customizability.^[^ ^42^, ^43^ ^]^ The pouch actuator has a structure consisting of two thin films and operates by inflating the pouch due to fluid pressure inside. In this research, the pouch actuator is driven by our pump, which overcomes the conventional issues of bulkiness, rigidity, and noise limitations, and can be operated continuously by allowing for dielectric breakdown. In addition, both the pump and linear pouch actuator can be fabricated by digital devices, and both are customizable. Therefore, the system can be expected to be used in a variety of applications. The configuration of this system is shown in **Figure** **5**a. A linear pouch actuator (1.5 g, 80 um thickness) is connected to a pump (100 pairs, 4 mm width) through a tube and filled with a dielectric liquid. The detailed design and fabrication method of the pouch actuator are shown in Figures S14 and S15 (Supporting Information). The combined weight of the linear pouch actuator and pump is about 10 g and is lightweight. The linear pouch actuator was operated by applying voltage while the pump was twisted. The fluid pressure generated by the pump deformed and drove the linear pouch actuator (Figure 5b; Movie S6, Supporting Information). After the dielectric breakdown, strokes were obtained again. Our pumps are also capable of operating sufficiently with a load‐mounted linear pouch actuator (Figure 5c–e; Movie S7, Supporting Information). At no load, a maximum strain of 26 % and a strain of 5 % was achieved at 10 N (Figure 5f). Its response time is rather long at 20 s (Figure S16b, Supporting Information), but this is a sufficient timeframe for a biomimetic device. Table S3 (Supporting Information) shows performance comparable to that of previous linear pouch actuators. Our actuator's force at 5% strain per size outperforms those driven by methods except mechanical pump‐driven. Additionally, our pouch actuator's maximum strain per size surpasses that of other actuation methods previously proposed. Furthermore, our pouch actuators are a promising option for situations where compactness is required.

**Figure 6:**
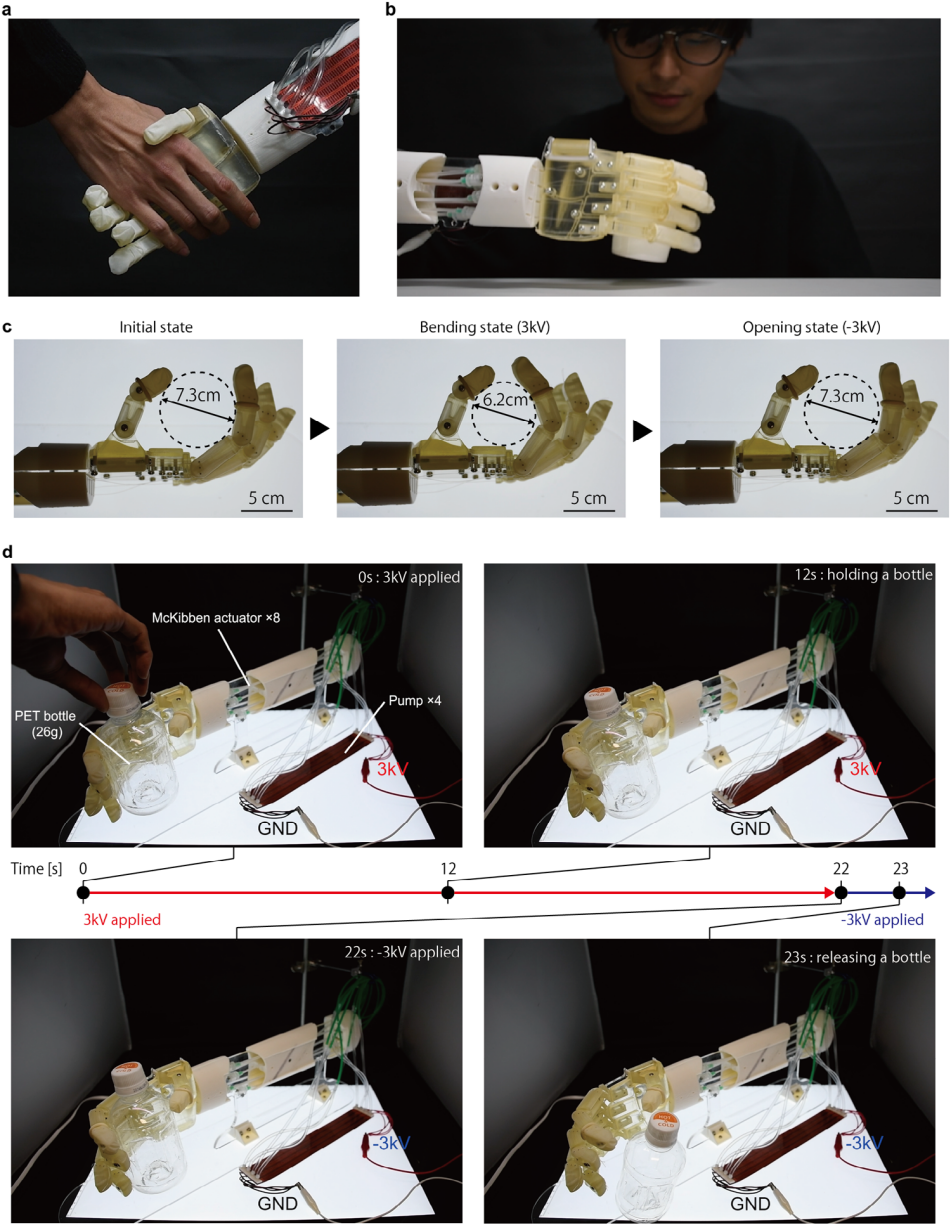
A prosthetic hand driven by four pumps. a) Handshaking and b) grasping a cup were demonstrated by a prosthetic hand with McKibben artificial muscles driven by four pumps (100 pairs, 2 mm width). c) Movement of the prosthetic hand when ±3kV was applied to the pumps. d) Photograph of the sequence of grabbing and releasing a plastic bottle.

McKibben artificial muscles are used in many musculoskeletal robots owing to their properties similar to natural skeletal muscle in terms of contraction ratio and generated force.^[^ ^44^, ^45^ ^]^ In the previous study, EHD pumps have been used to drive McKibben artificial muscles, providing a quiet and compact system.^[^ ^46^ ^]^ However, the pumps lacked generated pressure, limiting the operation of the McKibben artificial muscle. Here, we developed a prosthetic hand using the McKibben artificial muscles and our pumps (100 pairs, 2 mm width) that produced maximum pressure and successfully operated it (Movie S8, Supporting Information). The system configuration of the developed prosthetic hand is shown in Figure S17 (Supporting Information). Two McKibben artificial muscles are connected to both ends of the pump. When one McKibben artificial muscle is activated, the other acts as a tank and supplies dielectric liquid. This prosthetic hand can even perform daily activities such as shaking hands (**Figure** **6**a) and drinking a cup of water (100g) (Figure 6b). This prosthetic hand is actively driven in the open and closed directions by switching the polarity of the pump (Figure 6c). The largest circumscribed circle within the index finger to thumb range changes from 7.3 cm initially to 6.2 cm when 3 kV is applied, and the hand closes. The hand opens and returns to 7.3 cm when 3 kV is applied. This bidirectional motion allows the hand to grasp and release a 26 g plastic bottle (Figure 6d).

**Figure 7:**
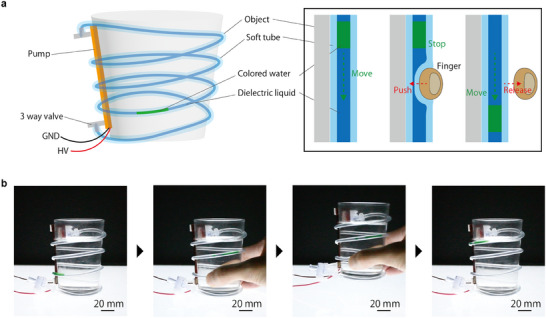
A tube‐format display with colored water transported by a mounted pump. a) This display is composed of a tube, a pump (10 pairs, 4 mm width), colored water, and dielectric liquid. The tube can be attached to complex object shapes by wrapping it around, and the information is presented as the colored liquid is transported through the tube. Our pump can transport the colored water indirectly, thanks to the separation of the colored water and the dielectric liquid. Furthermore, the interaction with a human is expressed by the speed of transport of the colored water, thanks to the softness of the tube. b) These systems, including the pump, are flexible and can be mounted to objects with curved surfaces, such as cups. When a person grabs the cup around which the tube is wrapped, the speed of colored water transport decreases, visually indicating grasping information.

In this study, we propose a novel tube format display that works by our pump (**Figure** **7**a; Movie S9, Supporting Information). As an example, we have realized an interaction that uses the flow of colored water in a tube attached to the surface of a glass cup, where the velocity of the fluid decreases in response to the contact pressure when the cup is grasped (Figure 7b). Taking advantage of the quiet operation and flexibility of our pump, this can easily be integrated into everyday spaces. The technology enables a variety of information presentations and new forms of interaction with the user, leading to a new realm of 3D dynamic display that goes beyond conventional 2D displays and projection mapping. The display consists of a pump, a tube, a dielectric liquid (transparent) with EHD operable, and conductive colored water (green) as the display medium. The information is presented by the movement of colored water in a tube wrapped around an object. Thanks to the combination of the physical properties of the liquids, the colored water is not mixed with the dielectric liquid, so the colored water is transported indirectly by transporting the dielectric liquid with our pump. By switching the polarity of the voltage at the appropriate time, continuous transport of colored water through the tube is possible. Although the risk of dielectric breakdown during the inflow of conductive liquid exists, this pump's resilience maintains stable operation without permanent failure. Furthermore, the possibility of new information presentation and user interaction is opened up by capturing the fluid transport velocity fluctuation caused by the user's contact with the tube as an interaction. In particular, the velocity fluctuation caused by grasping the cup is expected to be used as a tool to indicate the strength of hand force as feedback in rehabilitation situations.

## 3 Conclusion

Our flexible EHD pump overcomes its fragility, by incorporating resilience to dielectric breakdown. This advancement removes rigid, bulky, noisy, mechanical pumps from soft fluid systems and opens a new door to the human‐machine interface. Whereas past EHD pumps treat dielectric breakdown as a problem to be avoided, our pump is designed to allow it. This approach allows us to deal with the disturbances expected in application scenarios and to meet the growing demand for robust and adaptive devices in human‐centered applications.

Our pump is itself passively resilient, i.e., its novel electrode structure can maintain the insulation necessary for EHD pumping without an external active mechanism. In the experiments, we have verified resilience to a variety of scenarios in which dielectric breakdown can occur, including repetitive and prolonged high‐voltage and contaminations by bubbles and conductive liquids. To return to a pumpable state again, the inside of the pump must be filled with dielectric liquid. We built an active resilient system with two pumps arranged in series. When one pump experiences a dielectric breakdown, the other pump automatically activates, fills the system with dielectric liquid, and automatically recovers to a pumpable state. These two strategies, passive resilience of the pumps alone and active resilience of the system design reinforce human‐machine communication through the soft fluid system.

Furthermore, our EHD pumps are resilient yet structurally simple and can be fabricated with digital technology. This allows for customization and expansion, which is useful in the context of various human‐machine interfaces. Our pumps can also be operated in a bent or twisted position. This adaptability is important for integration into space‐limited environments typical of human‐machine applications. The pump structure was designed for moderate bending, which can be acceptable for some wearable applications. The pump principle itself can be applied to a wide range of HMI, yet the current embodiment of the pump does not work well at more extreme bending. The reason for the decrease in performance with curvature is that the channel deforms due to buckling, bringing its geometry far from the design point. To solve this limitation, the mechanics of the pump body should be studied under bending and both geometry and materials should be optimized for working at high curvatures. Additionally, incorporating external pressure and flow sensors to enable feedback control of input voltage could be a viable solution. This enhancement could significantly extend the usability and robustness of our pump in a wider range of HMI applications.

In pursuing further advancements, we recognize the need to explore new materials, designs, and manufacturing methods for EHD pumps. A significant challenge is electrode and fluid performance deterioration after breakdown, impacting pumping capability. Future applications will require sensing capabilities and controlled voltage inputs. Improvements are also needed to expand pumping performance, particularly flow rate. Current limitations, such as channel width constraints affecting flow rate, can be addressed by parallel pump configurations or new electrode structures. Additionally, our research will focus on enhancing the active resilience of our EHD pump system to safeguard against failure scenarios robustly, ensuring reliable and efficient operation across all conditions. We plan to integrate additional backup pumps and implement advanced control algorithms, which will allow for dynamic role swapping and improved management of simultaneous breakdowns. Lastly, Ensuring safety, particularly for applications within HMI, is paramount. In response to this need, our future research will focus on enhancing the overall insulation of the circuitry to protect users from the potential dangers of high currents resulting from dielectric breakdowns.

## 4 Experimental Section

### Dielectric Liquid

Novec 7300 (3M) was used as the dielectric liquid. EHD pumping requires low conductivity (<10^−7^S m^−1^), high dielectric withstand voltage, and high dielectric constant. Novec 7300 has a conductivity of 10 ^−9^S m^−1^, a dielectric withstand voltage of 5‐6 kV (gap: 0.5 mm), and a dielectric constant of 6.1. It also has a boiling point of 76 °C, high thermal conductivity, ultra‐low toxicity, zero ozone depletion potential, zero flash point, and non‐flammability, making it suitable for human interaction applications.

### Experimental Setup for Pump's Evaluations

The experimental setup shown in the figure was used to evaluate the pumps. A pressure sensor and flow rate sensor were used to measure the pump's output. A high‐voltage amplifier was used to supply voltage, and a function generator was used to generate arbitrary voltage waveforms. For the measurement of current, a resistor was placed in series with the GND side of the pump and the voltage across the resistor was measured, which was converted to current. Each output and input was measured via an oscilloscope.

### Repeated Dielectric Breakdowns Test

To verify the resistance of these pumps to electrical breakdowns, intentionally caused electrical breakdowns by increasing the applied voltage. A constant voltage was applied for 10 s, and if no electrical breakdown was observed, the voltage was increased by 0.5 kV until an electrical breakdown occurred. During this process, the maximum voltage that could be applied and the maximum pressure produced were plotted in the figure. In this test, the bubbles produced by the electrical breakdown were removed after the electrical breakdown, and 100 electrical breakdowns were verified. For comparison, a comb electrode pump was also evaluated, which is composed of the same material as our pump.

## Conflict of Interest

The authors declare no conflict of interest.

## Supporting information

Supporting Information

Supplemental Video 1

Supplemental Video 2

Supplemental Video 3

Supplemental Video 4

Supplemental Video 5

Supplemental Video 6

Supplemental Video 7

Supplemental Video 8

Supplemental Video 9

## Data Availability

Research data are not shared.
