# Supplementary material for: Resilient and Flexible Electrohydrodynamics Pumps for Human–Machine Interfaces
Source: Adv Sci (Weinh). 2025 Apr 16;12(22):2416502. doi: 10.1002/advs.202416502 (PMC12165118; doi:10.1002/advs.202416502)
Supplement: Supplementary file 1 — Supporting Information [file ADVS-12-2416502-s002.pdf]

ADVANCED SCIENCE
Open Access

Supporting Information

for *Adv. Sci.*, DOI 10.1002/advs.202416502

Resilient and Flexible Electrohydrodynamics Pumps for Human–Machine Interfaces

*Yu Kuwajima, Amr Marzuq, Soraya Segawa, Yuya Yamaguchi, Yuhei Yamada, Takafumi Morita, Katrene Morozov, Haruto Iwasaki, Sota Suzuki, Hiroyuki Nabae, Vito Cacucciolo, Naoki Hosoya, Yasuaki Kakehi and Shingo Maeda**

Supplementary information

Resilient and Flexible Electrohydrodynamics Pumps for Human-Machine Interfaces

Yu Kuwajima[1,6], Amr Marzuq[1], Soraya Segawa[2], Yuya Yamaguchi[2], Yuhei Yamada[3], Takafumi Morita[4], Katrene Morozov[5], Haruto Iwasaki[1], Sota Suzuki[1], Hiroyuki Nabae[1] Vito Cacucciolo[6], Naoki Hosoya[2], Yasuaki Kakehi[4], Shingo Maeda[1,3*]

[1]Department of Mechanical Engineering, Institute of Science Tokyo
2-12-1, Ookayama, Meguro-ku, Tokyo, 152-8550, Japan

[2]Department of Engineering Science and Mechanics, Shibaura Institute of Technology
3-7-5, Toyosu, Koto-ku, Tokyo, 135-8548, Japan

[3]Research Center for Autonomous Systems Materialogy (ASMat), Institute of Innovative Research, Institute of Science Tokyo
4259, Nagatsuta-Cho, Midori-Ku, Yokohama, Kanagawa, 226-8501, Japan

[4]The University of Tokyo
7-3-1, Hongo, Bunkyo-ku, Tokyo, 113-8654, Japan

[5]Department of Physics, University of California, Santa Barbara
Broida Hall, Santa Barbara, CA 93106, USA

[6]Department of Mechanics, Mathematics and Management (DMMM), Politecnico di Bari, Bari, Italy.

*Corresponding author. E-mail: maeda.s.ao@m.titech.ac.jp (S.M)

This PDF file includes

Supplementary note 1: Pump's design and fabrication

Supplementary note 2: Experimental setup for pump's evaluation

Supplementary note 3: Evaluation of resilience to dielectric breakdown

Supplementary note 4: Evaluation of pumping performances

Supplementary note 5: Demonstration of linear pouch actuator

Supplementary note 6: Demonstration of Mckiben muscles-driven prosthetic hand

Supplementary note 7: Demonstration of tube-formated display

Supplementary Figures 1-17

Supplementary Tables 1-3

Legends for movie S1-S9

Supplementary note 1: Pump's design and fabrication

Pump's design

The performance of electrohydrodynamic (EHD) pumps is primarily influenced by the strength of the electric field. The stronger the electric field along the flow direction, the higher the pressure and flow rate. To prevent potential pump damage from dielectric breakdowns, this study explores the use of a diagonal electrode arrangement. In this section, we undertake a comprehensive numerical analysis to find the best design for achieving the highest pressure density and fluidic power density.

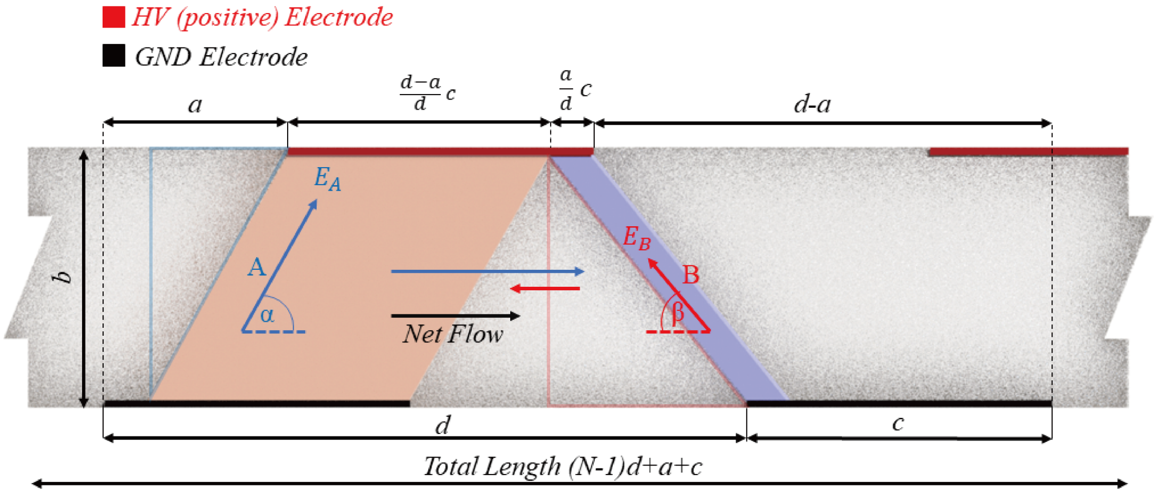

Fig. S1 The pump cross-section geometry

Theory

Fig.S1 illustrates the design of the diagonal electrode structure in our pumps. Here, we maximize the pressure difference induced by the pump divided by the area of the channel. Denoting the pressure difference as P [Pa] and the area as A [m^2], the optimization function becomes

$$X = P/A \tag{1}$$

Pressure distribution in the EHD pump is complex and difficult to calculate. For simplicity, we adopt a rough estimation for the pressure difference, Δp, on the dependence on the strength of the electric field, E [V/m], and the constant, K, as

$$\Delta p = KE^2 \tag{2}$$

The electric field formed by the electrodes in the geometry is also very complex. Here, we adopt a rough assumption that the electric field is approximated by two uniform ones in a unit structure each represented by pink and purple domains in Fig.S1. The direction of the electric field in the pink domain is denoted by A and the angle to the direction of net flow is α, and those for the purple domain are B and β, respectively. The electric field in the A direction can be characterized as follows:

$$E_A = V/L_A \tag{3}$$

Where V is the electric voltage [V]. From the pump geometry:

$$L_A = \sqrt{(a - \frac{a}{d}c)^2 + b^2} \tag{4}$$

$$E_A = \frac{V}{\sqrt{(a - \frac{a}{d}c)^2 + b^2}} \tag{5}$$

$$P_A = K\frac{V^2}{(a-\frac{a}{d}c)^2+b^2} \tag{6}$$

Since pressure in the diagonal direction is dispersed, we assumed that the pressure in the horizontal direction is dominant.

$$P_{A_H} = K\frac{V^2}{(a-\frac{a}{d}c)^2+b^2}cos(\alpha) \tag{7}$$

The same in the B direction.

$$E_B = V/L_B \tag{8}$$

$$L_B = \sqrt{(d-a-c+\frac{a}{d}c)^2+b^2} \tag{9}$$

$$E_B = \frac{V}{\sqrt{(d-a-c+\frac{a}{d}c)^2+b^2}} \tag{10}$$

$$P_B = K\frac{V^2}{(d-a-c+\frac{a}{d}c)^2+b^2} \tag{11}$$

$$P_{B_H} = K\frac{V^2}{(d-a-c+\frac{a}{d}c)^2+b^2}cos(\beta) \tag{12}$$

The pressure in the flow direction generated within one unit depicted in Fig.S1 is given by:

$$P_{unit} = P_{A_H} - P_{B_H} \tag{13}$$

Therefore, the pressure in the flow direction generated within N units is given by:

$$P_{total} = NP_{A_H} - (N-1)P_{B_H} \tag{14}$$

$$P_{total} = K\frac{NV^2}{(a-\frac{a}{d}c)^2+b^2}cos(\alpha) - K\frac{(N-1)V^2}{(d-a-c+\frac{a}{d}c)^2+b^2}cos(\beta) \tag{15}$$

$$P_{total} = KV^2\left[\frac{Ncos(\alpha)}{(a-\frac{a}{d}c)^2+b^2} - \frac{(N-1)cos(\beta)}{(d-a-c+\frac{a}{d}c)^2+b^2}\right] \tag{16}$$

Where,

$$cos(\alpha) = \frac{a-\frac{a}{d}c}{\sqrt{(a-\frac{a}{d}c)^2+b^2}} \tag{17}$$

$$cos(\beta) = \frac{d-a-c+\frac{a}{d}c}{\sqrt{(d-a-c+\frac{a}{d}c)^2+b^2}} \tag{18}$$

Therefore;

$$P_{total} = KV^2\left[\frac{N(a-\frac{a}{d}c)}{\left[(a-\frac{a}{d}c)^2+b^2\right]^{3/2}} - \frac{(N-1)(d-a-c+\frac{a}{d}c)}{\left[(d-a-c+\frac{a}{d}c)^2+b^2\right]^{3/2}}\right] \tag{19}$$

The area of the channel is;

$$A = [(N-1)d+a+c]b \tag{20}$$

Therefore the optimization equation is;

$$X = \frac{KV^2}{[(N-1)d+a+c]b}\left[\frac{N(a-\frac{a}{d}c)}{\left[(a-\frac{a}{d}c)^2+b^2\right]^{3/2}} - \frac{(N-1)(d-a-c+\frac{a}{d}c)}{\left[(d-a-c+\frac{a}{d}c)^2+b^2\right]^{3/2}}\right] \tag{21}$$

The function that describes the power density Y can be obtained by the below assumption:
First: The relation between pressure P and flow rate Q [L/min] is linear [1]. As shown in Fig.S2, the equation that describes the linear relation is:

$$Q = -\frac{Q_{max}}{P_{max}}P + Q_{max} \tag{22}$$

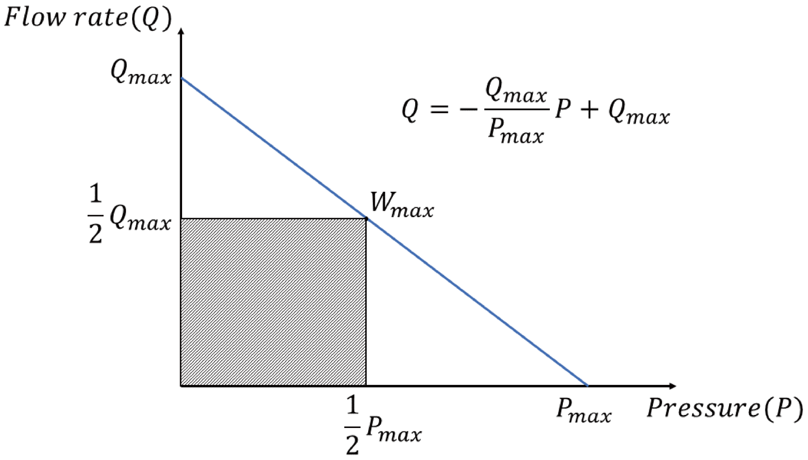

Fig. S2 The Pressure Flow rate curve.

Where;

$$0 \le Q \le Q_{max} \tag{23}$$

$$0 \le P \le P_{max} \tag{24}$$

The fluidic power $W[W]$ can be obtained by:

$$W = PQ \tag{25}$$

and as described by the linear relation can be written as:

$$W = P(-\frac{Q_{max}}{P_{max}} P + Q_{max}) \tag{26}$$

At the point of maximum power:

$$\frac{\partial W}{\partial P} = 0 \tag{27}$$

As a result:

$$P = P_{max}/2 \tag{28}$$

By substitution in the equation 22, Q for the maximum power can be obtained as:

$$Q = Q_{max}/2 \tag{29}$$

As a result W_{max} will be:

$$W_{max} = \frac{P_{max} Q_{max}}{4} \tag{30}$$

The Q_{max} needs to be eliminated to have a power function in terms of pressure
Second: The Hagen–Poiseuille law concerning Reynolds number is used to have the relation between the pressure and flow rate as follows:

$$\Delta P = \frac{8\pi\mu H}{S^2} Q \tag{31}$$

where μ is the dynamic viscosity of the liquid, H is the channel length, and S is the the cross-sectional area of channel. As the channel dimensions are constant the equation can be written as:

$$\Delta P = RQ \tag{32}$$

Where R is constant. For the maximum flow rate

$$P_{max} = RQ_{max} \tag{33}$$

$$Q_{max} = P_{max}/R \tag{34}$$

From equation 1:

$$P_{max} = XA \tag{35}$$

The optimization function for power density Y can be obtained by:

$$Y = W_{max}/A \tag{36}$$

By substitution from equations 30,34, and35 Y will be:

$$Y = \frac{A}{4R}X^2 \tag{37}$$

To conclude, the power density has a parabolic relation concerning the pressure density. As a result at the point of maximum pressure density, the power density must be the maximum.

$$Y_{max} = \frac{A}{4R}X_{max}^2 \tag{38}$$

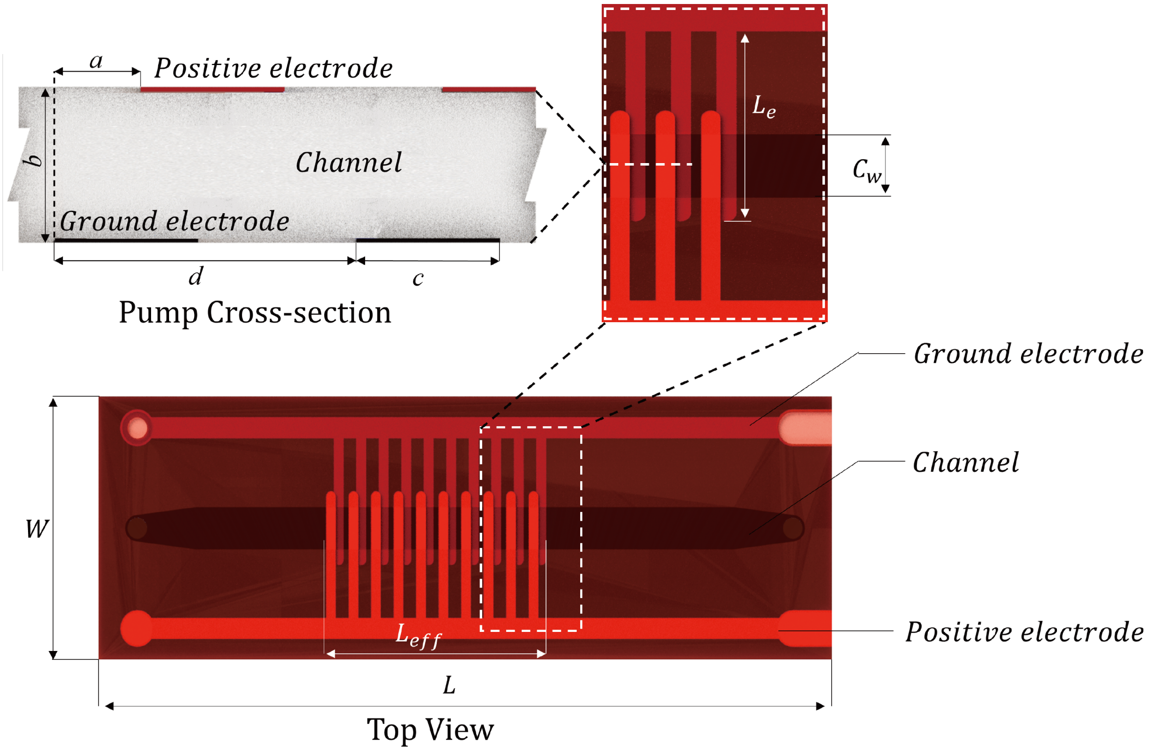

Fig. S3 The pump geometry.

Calculation and simulation for optimizing the design

In this study, we optimized a and d from calculations and simulations of equation 21: a two-dimensional model was built to match the dimensions of an actual 10-pair pump using COMSOL, and the electric field was simulated (Fig.S4a). Color change indicates an electric field in the flow direction. Here, the calculations and simulations were performed using N=10, K=1, V=1 [kV], b=0.5 [mm], and c=1 [mm] as the main parameters. Fig.S4b,c shows X (color change) for a, d obtained from calculations and simulations. The calculations show that the X peak was at a=0.5 [mm] and d=2.8 [mm] in the range $d > c$ and $d > a$ where the geometry is valid(Fig.S4b). Simulation results show peak X at a=0.5 [mm] and d=2.1 [mm](Fig.S4c). The slight deviation in the optimum value of d may be due to the fact that the calculation considers only the forward electric field between facing electrodes and the reverse electric field between adjacent electrodes. In reality, more electrodes should affect the electric field. Nevertheless, the calculations and simulations are in approximate agreement. This allows a simple estimate of the optimal value from the calculation. The usefulness of similar calculations and simulations, albeit with different pump geometries, has been demonstrated in pump evaluation experiments[2]. In this study, the optimal values a=0.5 [mm] and d=2.1 [mm] obtained from the simulation were used to design the pump.

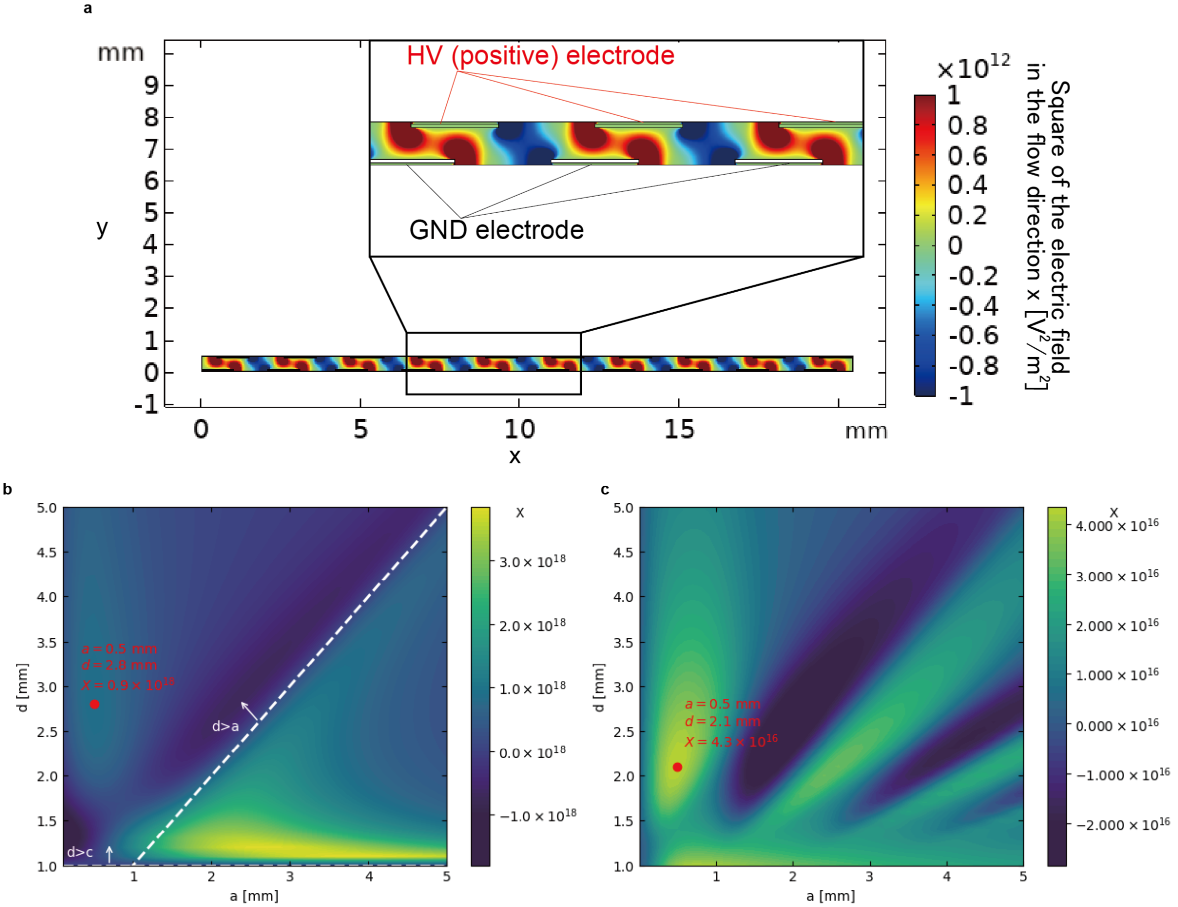

Fig. S4 Optimization of pump structure. **a** Simulated electric field in the flow direction in the pump. Optimal geometry values are derived by **b** calculation and **c** simulation.

Geometry design

Fig.S3 depicts the design of the diagonal electrode configuration employed within the examined EHD pumps. The top view of the illustration outlines key dimensions pertinent to the pump, such as L representing the pump's length, W denoting the pump's width, and L_{eff} is the effective length of the pump. Within the zoomed-in section, specific dimensions are highlighted, including the channel width

c_w and the electrode length L_e. The pump's cross-sectional representation elucidates the fundamental electrode structure: the positive electrode situated at the top, the ground electrode positioned at the bottom, and the channel existing between them. Notably, the channel height b is set at a constant value of 0.5 [mm], mirroring the thickness of the VHB layer used for the channel layer. The electrode width c remains fixed at 1 [mm], while a represents the pair shift, maintaining a consistent value of 0.5 [mm], and d corresponds to the normal shift, measured at 2.1 [mm].

Table S1 demonstrates the design parameters' values in [mm] for the different pumps used in this work.

Table S1: Geometry design parameters.

Pump name	N	c_w	L_e	L	W	L_{eff}
N10, c_w4	10	4	10	75	25	20.4
N50, c_w4	50	4	10	135	25	104.4
N100, c_w2	100	2	4	240	25	209.4
N100, c_w4	100	4	6	240	25	209.4
N100, c_w8	100	8	10	240	25	209.4
N100, c_w16	100	16	18	240	33	209.4

Fabrication Process

The fabrication process comprises two primary stages: material fabrication and assembly. Each stage involves distinct procedures as elaborated below.

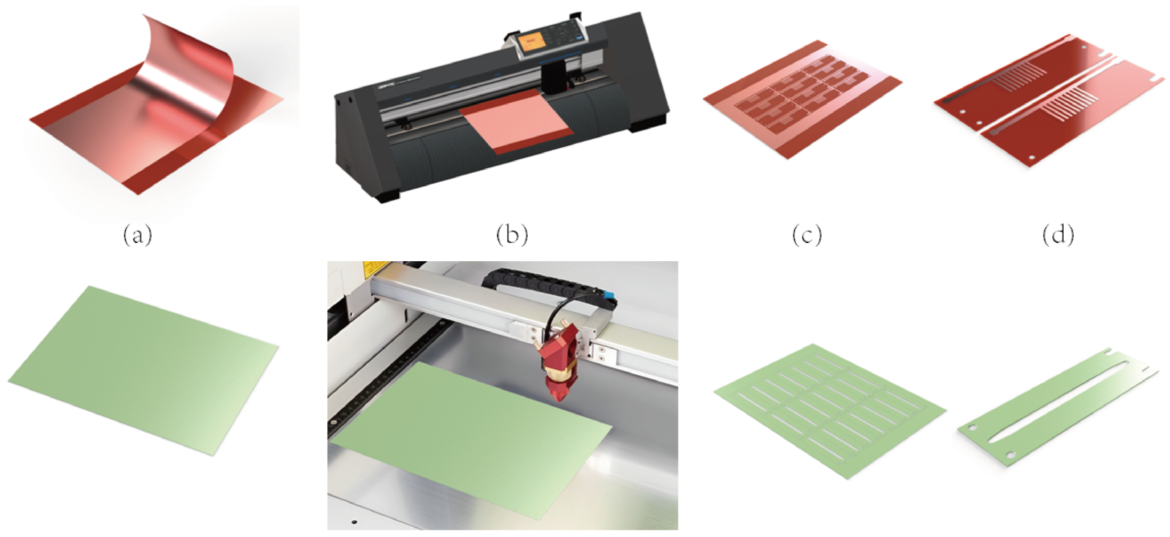

(a) (b) (c) (d) (e) (f) (g) (h)

Fig. S5 The fabrication process.

Material Fabrication Stage

Fig.S5 depicts the sequential steps involved in the fabrication process in detail as follows: The initial step involves merging the polyamide sheet (depicted in orange) with an adhesive copper foil, showcased in Fig.S5(a). Subsequently, a plotting cutter machine (CE7000-40, Graphtec) Fig.S5(b) is utilized to intricately cut the pump's design from both the copper foil and polyamide sheet. To cut copper foil without cutting the polyamide two different cutting characteristics are used. The copper is cut using a force of 12 [N] with a cutting speed of 2 [cm/sec] in one shoot while a force of 38 N is used to cut the polyamide sheet with a speed of 1 [cm/sec] in three paths. The unneeded copper is pulled out

as shown in Fig.S5(c). This process yields the bottom and upper layers of the pump, illustrated in Fig.S5(d).
The middle layer which hosts the channel is a VHB (Y-4905J, 3M) adhesive soft acrylic material with a thickness of 0.5 [mm] and covered with plastic foil for both sides Fig.S5(e). The VHB layer is cut using a laser cutting engraver machine Fig.S5(f). After cutting, unwanted material is removed as shown in Fig.S5(g). The process ends up by the middle VHB layer as in Fig.S5(h).

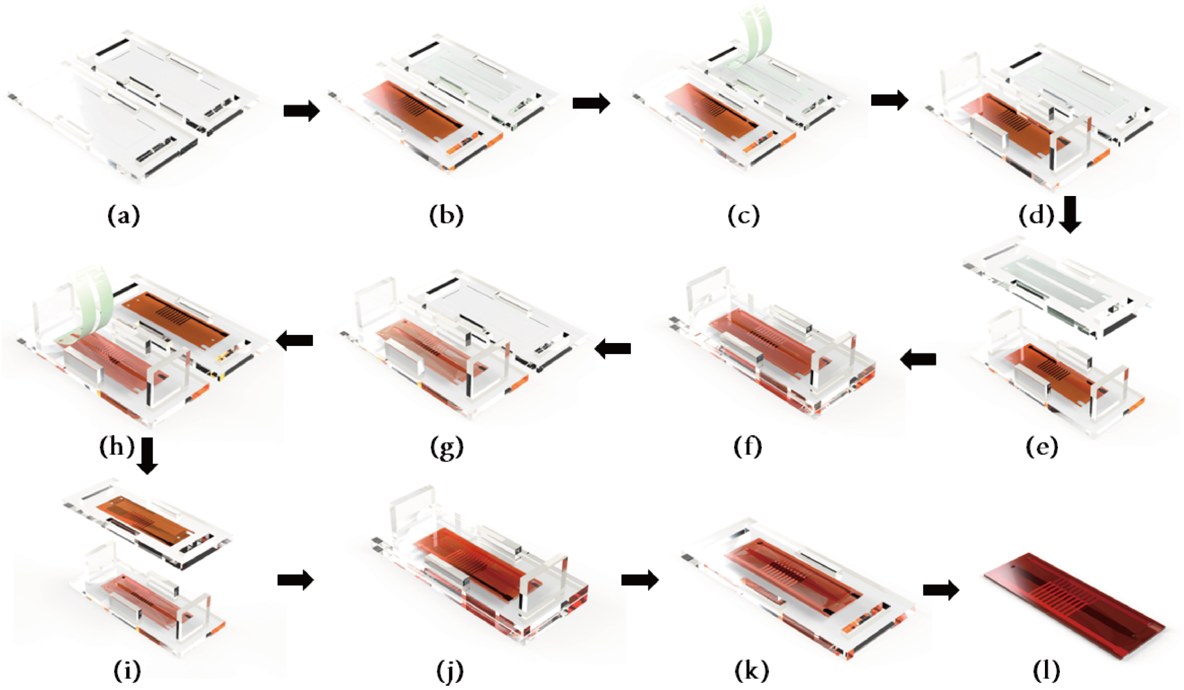

Fig. S6 The assembly process.

Assembly Stage

The subsequent phase involves the assembly process, where the pump is constructed by aligning its three layers: top, middle, and bottom. The assembly procedure is outlined in detail within Fig.S6:
Guided Molds:
To facilitate a reliable assembly process, two guided molds made of acrylic are employed (as depicted in Fig.S6(a)).
Layer Assembly Steps:
The bottom layer, consisting of polyamide with copper foil, is positioned within one of the molds(Fig.S6(b)). Simultaneously, the other mold holds the middle layer comprising the adhesive VHB along with the channel. The adhesive VHB layer, with a thickness of 0.5 mm and protective covers on both sides, has these covers removed just before the assembly process (Fig.S6(c)).
Two pairs of acrylic guides are utilized to align and place the two layers atop one another, ensuring proper positioning (Fig.S6(e), (f), and (g)). The subsequent step involves placing the top layer (polyamide with copper foil) within one mold, while the other mold contains the assembled bottom layer with the middle layer (Fig.S6(h)). Similar alignment guides are utilized to ensure proper alignment (Fig.S6(i), (j), and (k).
After the alignment process, the guides and acrylic molds are removed, resulting in the completion of the assembled pump (Fig.S6(l)).
This detailed assembly process involves the systematic arrangement of layers using guided molds and acrylic guides to ensure precise alignment, ultimately leading to the successful construction of the functional pump.

Supplementary note 2: Experimental setup for pump's evaluation

The experimental setup (Fig.S7) was used to evaluate the pump. The pump is connected to a chemical-resistant tube (LMT-55, Saint-Gobain) with an inner diameter of 2 mm and an outer diameter of 4 mm. The pump output was measured using a pressure sensor (GP-MO10, KEYENCE) and a flow rate sensor (FD-XS8, KEYENCE). A pinch valve (MPPV-4, Resolution Air) was used to regulate the flow rate. With the valve fully closed, maximum pressure was measured, and with the valve fully open, maximum flow rate was measured. A high-voltage amplifier (HEOPT-20B10, MATSUSADA Precision) was used to provide voltage, and a funk generator (Tektronix AFG1022) was used to generate arbitrary voltage waveforms. Current was measured by placing a 510ohm resistor in series with the GND side of the pump and measuring the voltage across the resistor, which was converted to current.

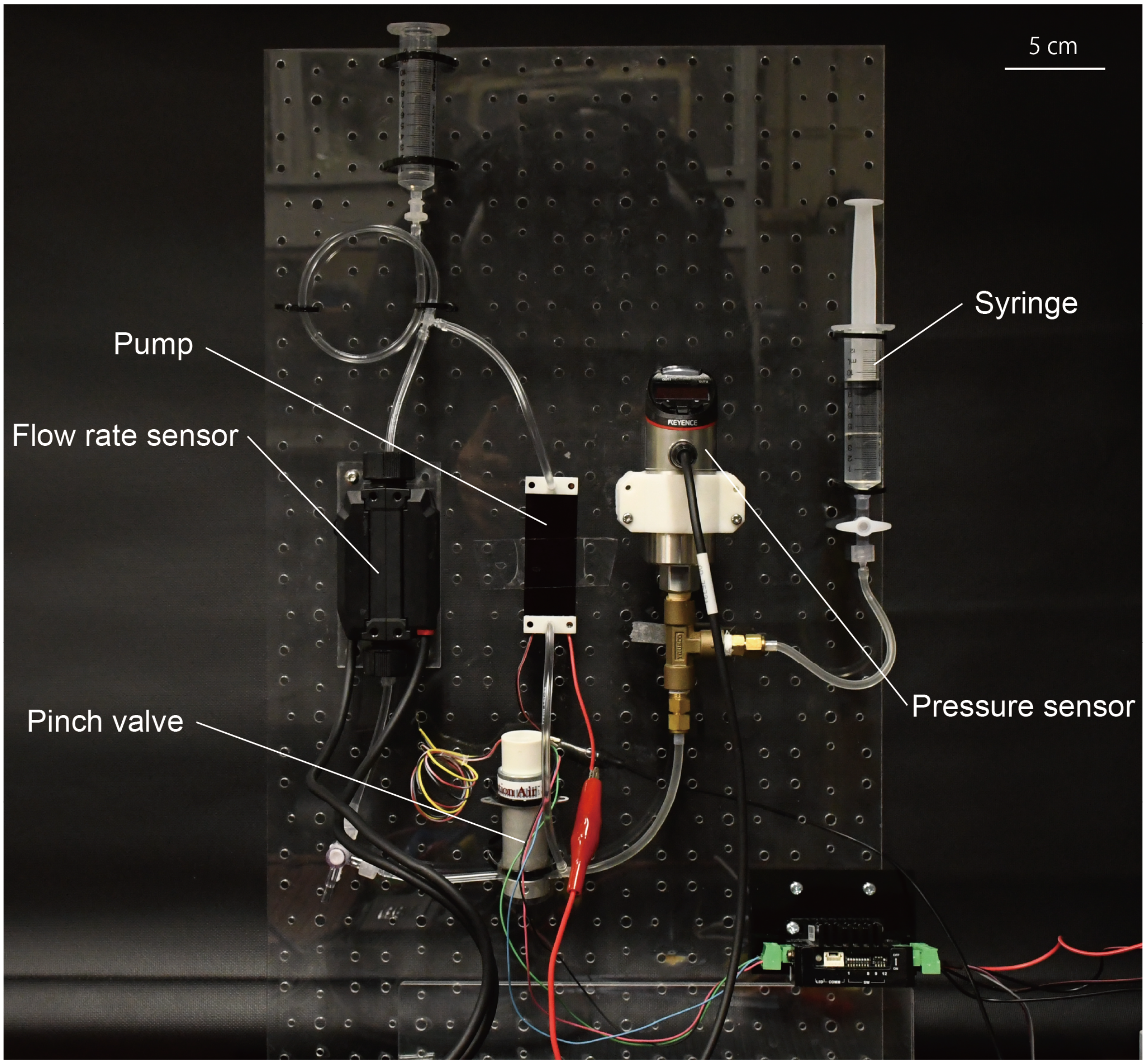

Fig. S7 Experimental setup for pumps

Supplementary note 3: Evaluation of resilience to dielectric breakdown

To verify the resilience of our pump (10 pairs, 4 mm width) to dielectric breakdown, we intentionally repeated the breakdown by increasing the applied voltage and measuring the breakdown voltage and maximum pressure. In this test, the dielectric liquid was first filled with fresh working fluid and then not replaced, removing only the bubbles generated during dielectric breakdown. A constant voltage was applied for 10 seconds, and the voltage was increased by 0.5 [kV] increments until breakdown occurred (Fig.S9a). The breakdown voltage and maximum pressure obtained during this process are plotted in Fig. Up to 100 times breakdowns (Fig.S9b) were verified. The relationship between voltage and pressure for each representative cycle is shown in Fig.S9c. The relationship between voltage and pressure tends to converge with each increase in the number of dielectric breakdowns. Fig.S9d shows the top view of the pump after 100 times breakdowns. It is observed that the area near the electrode turns black. While this may have a performance impact on the pump, it does not indicate a permanent breakdown. For comparison, a comb electrode pump made of the same material as our pump was also evaluated (Fig.S8a). The pumps cannot apply voltage and generate pressure after one breakdown. This is thought to be due to the loss of insulation due to thermal damage to the substrate surfaces between the electrodes caused by the breakdown. This is also estimated by observation of the black areas formed on the electrode surfaces (Fig.S8b). After 100 cycles of dielectric breakdown, the working liquid was replaced and a constant voltage of 5 [kV] was applied for a total of 4 hours to measure pressure and number of breakdowns. In this test, the working fluid was replaced after 2 hours.

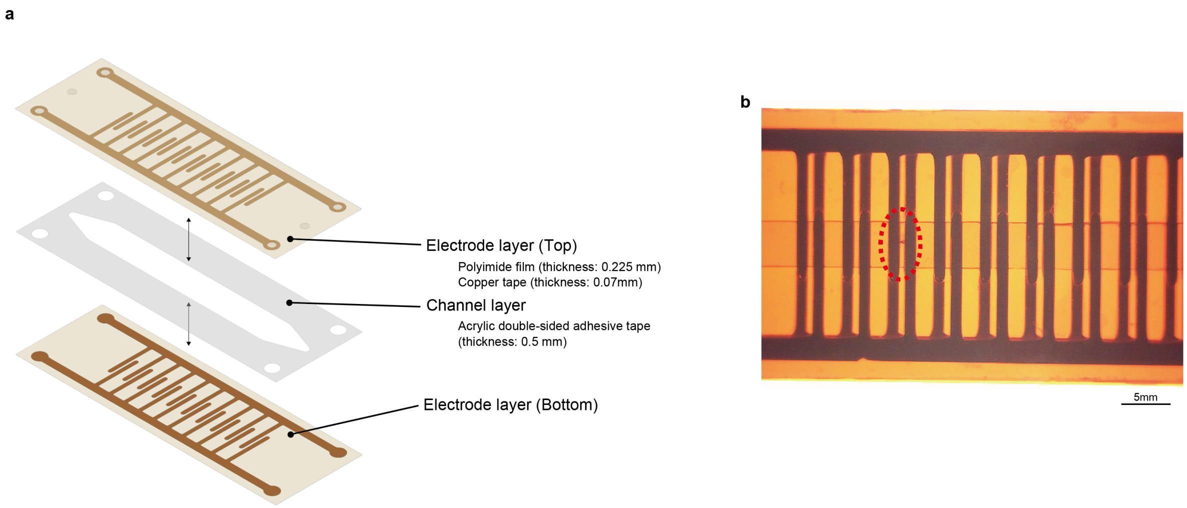

Fig. S8 Comb electrode EHD pump and its dielectric breakdown. a the pump's structure. **b** Top view of the pump after dielectric breakdown

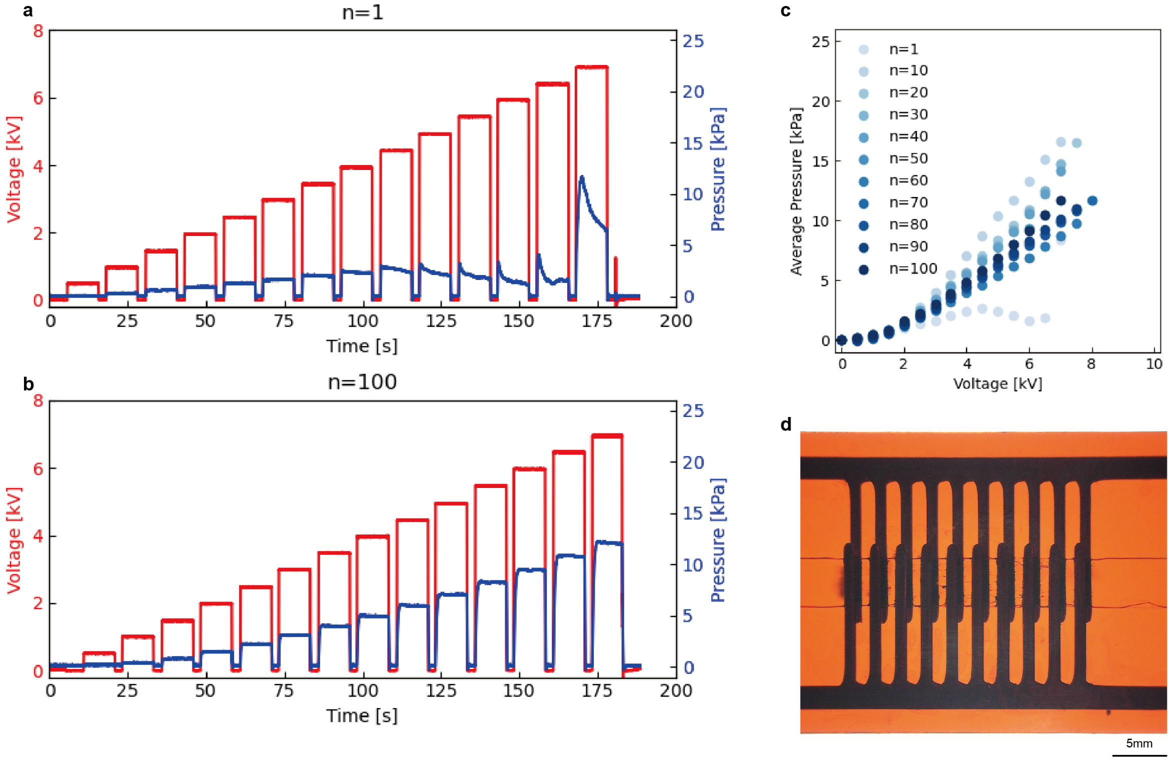

Fig. S9 Evaluation of Resilience to Dielectric Breakdown. a,b Time variation of voltage and pressure leading up to dielectric breakdown when the number of dielectric breakdowns N is 1 and 100.c Pump after 100 dielectric breakdowns.

Supplementary note 4: Evaluation of pumping performances

We investigated the voltage dependence of the pressure and flow rate of the various pumps. The voltage was increased in 0.5 [kV] increments until the breakdown occurred, and each voltage was applied for 1 minute. The average pressure and flow rate at each voltage and its standard deviation are measured and plotted (Fig.) Based on the simplified theory of rigid EHD pumps, the pressure should be proportional to the square of the voltage. However, this EHD pump tended to be proportional to the voltage from a certain threshold. This may be since the electrodes of our pump are arranged on different planes, and the deformation caused by the pressure reduces the generated electric field.

We then performed a fitting to characterize the generated pressure and flow rate versus voltage. The following are the equations we estimated.

$$P = eV^2(V \leq V_{threshold}) \tag{39}$$

$$P = fV + g(V > V_{threshold}) \tag{40}$$

$$Q = hV^2(V \leq V_{threshold}) \tag{41}$$

$$Q = iV + j(V > V_{threshold}) \tag{42}$$

The coefficients e, f, g, h, i, j were estimated by fitting, where the pressure or flow rate is proportional to the square of the voltage when the voltage is below a certain voltage threshold $V_{threshold}$ 39,41, and, where the pressure is proportional to the voltage when the voltage is above a certain threshold $V_{threshold}$ 40,42. The fitting was performed using the least-squares method in Python. There is a peak in the coefficient of determination when the voltage threshold is varied, and its value is greater than 0.9, suggesting the validity of this model equation. The fitting results when the coefficient of determination peaks are well matched, as shown in Fig.S10. The results of the fitting at the pump for each

geometry are shown in Fig.S11. The threshold pressure converted from the voltage using Equation 39 is about 10˜15 kPa and is constant regardless of the number of electrode pairs. The larger the channel width, the smaller its threshold pressure because it is more easily deformed.

The relationship between pressure and flow rate, as well as maximum fluid power and efficiency, were investigated by measuring pressure, flow rate, and current when the valve was allowed to transition from fully open to closed (Fig.S12a). As estimated by the equation 22, the relationship between pressure and flow rate was linear and well fitted (Fig.S12b) and its coefficient of determination was greater than 0.99 at any voltage (Fig.S12c).

The performance of the pumps was compared in Fig.S13 Since the maximum fluid power has not been reported for some previous EHD pumps, it was estimated using Equation37. This estimation is based on the linear pressure/flow relationship between our pumps and previous EHD pumps. Efficiency was also estimated from the estimated maximum fluid power and reported power consumption.

Evaluation of the pumping performance when bending the pump (100 pairs, 4mm width) was done for the experimental system shown in the figure. The pump was bent at the center and the pump was fixed so that the maximum radius of curvature was 10,6,4 [cm]. The maximum pressure and maximum flow rate were evaluated at 4 [kV].

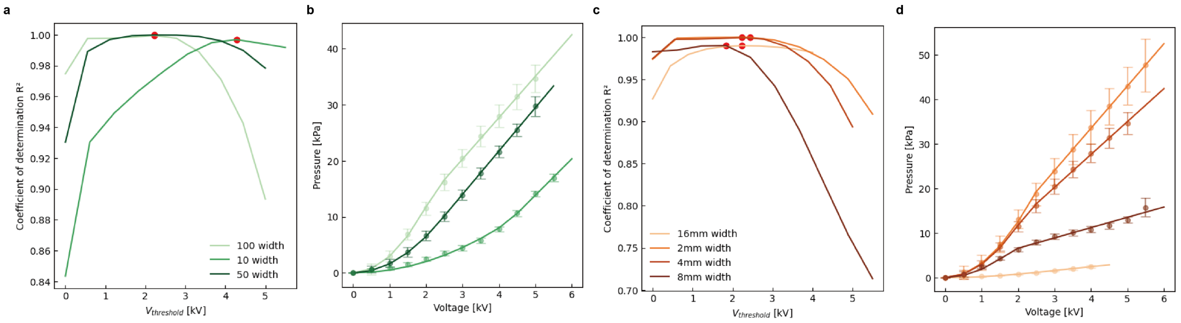

Fig. S10 Fitting results and determination of threshold. In the fitting of the relationship between applied voltage and pressure, **a** coefficient of determination for threshold voltage and **b** best fitting results. **c,d** the results in the fitting of the relationship between applied voltage and flow rate

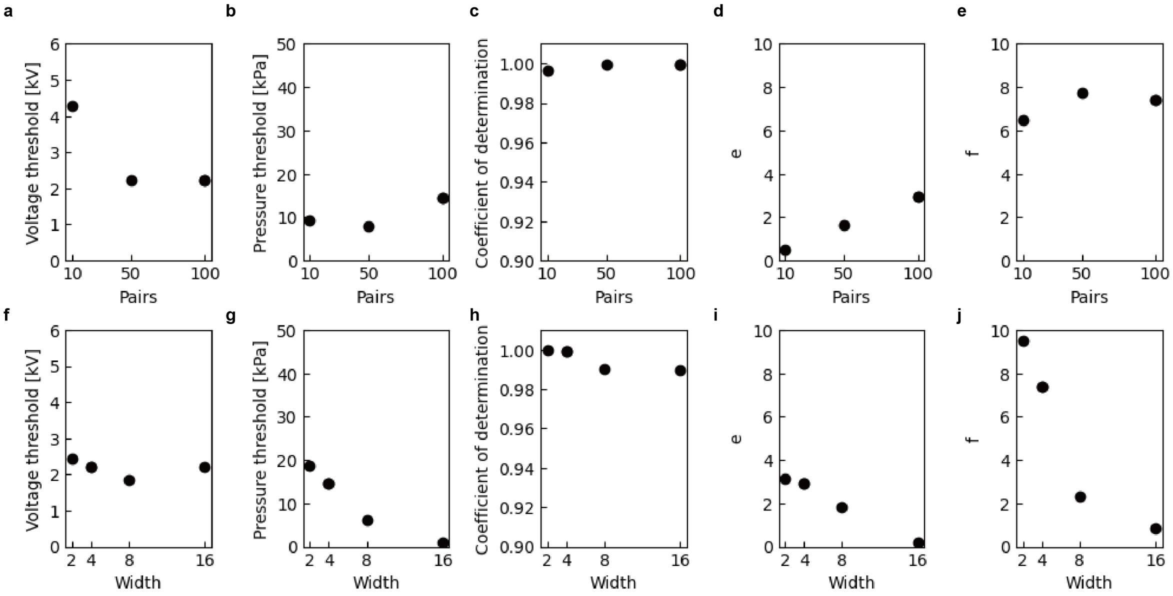

Fig. S11 Fitting results by pump's geometry.

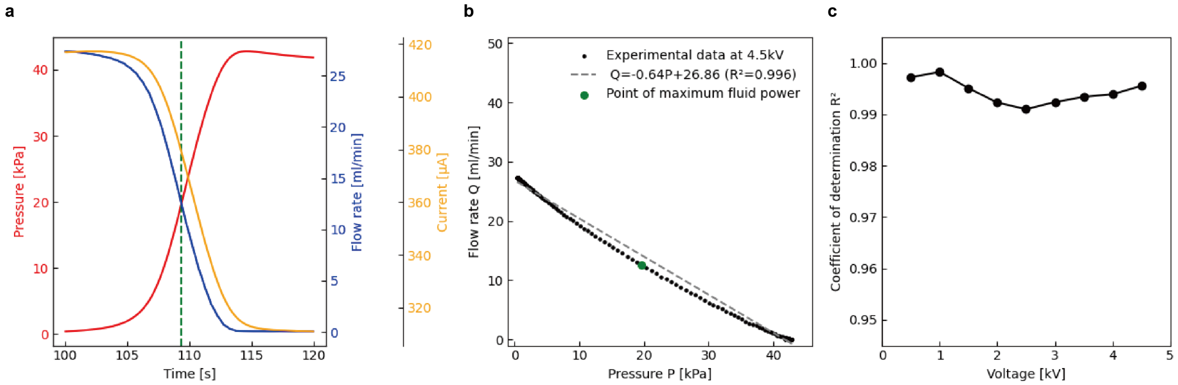

Fig. S12 The relationship between pressure and flow rate. **a** Pressure, flow rate, and current during valve closing. **b** Pressure and flow rate curve. **c** Coefficient of determination for linear fitting for each applied voltage. The green dashed lines and dots indicate where the fluid power is at its maximum. The gray dashed line is the result of fitting.

Table S2: Comparison between several EHD pumps' safety factors

References	Our Pump	[1]	[3]
Working Liquid	Novec 7300	Novec 7100	FC-40
Dielectric Strength [kV mm^{-1}]	>9.8	>9.8	>18.1
Safe Electric Field [kV mm^{-1}]	10	6	16
Reported Operation Time [h]	4	144	1
Safety Factor	1.0	1.6	1.1

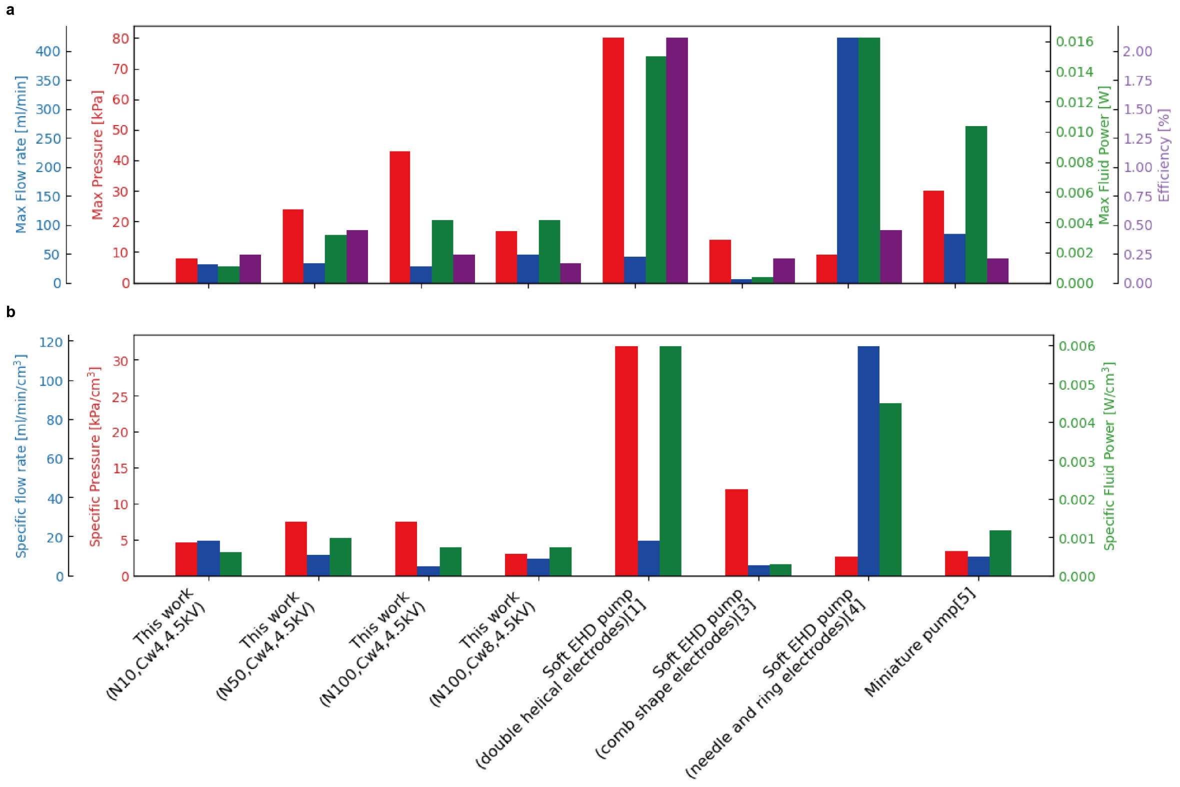

Fig. S13 Performance comparison between our pumps, previous soft EHD pumps[1, 3, 4] and commercially miniature pump[5]. **a** Absolute and **b** relative performances per size of each pump.

Supplementary note 5: Demonstration of linear pouch actuator

This study demonstrates the operation of a 50 x 65 x 0.08 [mm] linear pouch actuator with three chambers driven by an EHD pump (100 pairs, 4mm width). The detailed design of the linear pouch actuator is shown in Fig.S14. The film width $A = 50$ [mm], film height $B = 65$ [mm], chamber width $D = 40$ [mm], chamber height $L_0 = 10$ [mm], and channel width $w = 8$ [mm] connecting the three chambers. The chamber width aspect ratio $D : L_0$ =4:1 is large enough to reduce edge effects due to constrained sides. The width of the tube was designed to be at least 20 % of the w-channel width D to prevent pinching, which prevents fluid from reaching the very bottom of the tube [6]. The contraction force of the linear pouch actuator is proportional to the width D and the length L_0. The amount of deformation increases as the number of chambers and its vertical width L_0 is increased. The design can be adapted to the application.

The linear pouch actuator developed in this study is made of inexpensive materials, and its design can be freely customized by digital fabrication devices. The linear pouch actuator consists of two sheets of 40 [µm] thick polyethylene film (L-8, SEISANNIPPONSHA LTD). Connectors fabricated by a 3D printer (Formlab 3) and tubing with an outer diameter of 4 [mm] and an inner diameter of 2 [mm] (LMT-55, Saint-Gobain) were used as components connecting the linear pouch actuator and the pump. Fig.S15A: Polyethylene film was first cut into two pieces by a laser plotting cutter (TROTEC Speedy100). Its processing parameters are power of 25%, speed of 0.5%, and frequency of 2000 [Hz]. Fig.S15B: Next, a CNC heat sealer (CNC3018 Pro Max, MOSTICS) was utilized to thermo-compress the two pieces of polyethylene film at 270 [°C]. The film was then cut into two pieces. One part was not thermo-compressed to insert the connector; elastic PDMS was used as a foundation to allow for Z-axis errors. Its top surface was covered with a 25 [µm] thick polyimide film (100H-20 mm-20 m, DU PONT-TORAY CO., LTD.) to prevent melting of the polyethylene film due to excessive heating. The sealing width b is 2 [mm] to prevent leakage of the working liquid. Fig.S15C: Next, the connector was attached and the tube was installed. Finally, it was completely sealed with a heat sealer (FS-215, FUJIIMPULSE).

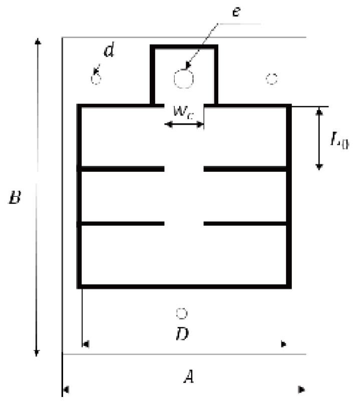

Fig. S14 Design of linear pouch actuator. The thin black line indicates the outer frame and the thick black line indicates the bonded area by thermo-compression.

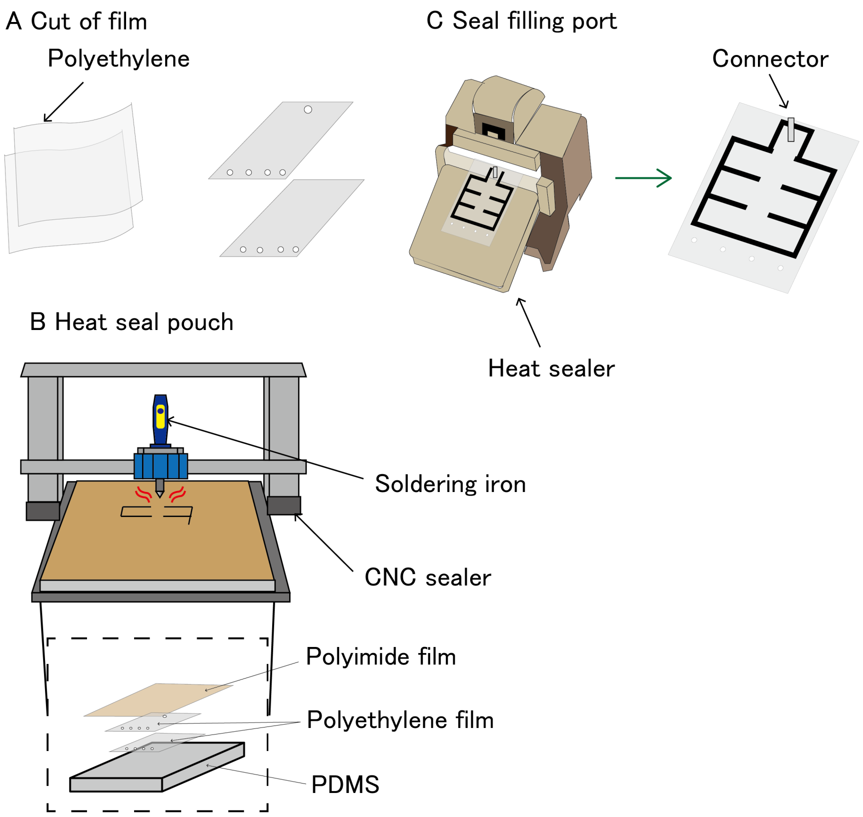

Fig. S15 Fabrication of linear pouch actuator.

The performance of our linear pouch actuator was evaluated in the experimental system shown in Fig.S16a. The linear pouch actuator is connected to a component with a marker to track deformation and a hole to hang a weight. A camera (DINOAM4113T, AnMo Electronics Corp.) was used to capture images of the markers, and video analysis software (Kinova) was used to analyze the deformation volume ($3(L_0 - L)$). Pressure was measured with a pressure sensor (AP-10SK, KEYENCE), and its data acquisition started simultaneously with the video recording.

The pressure and the deformation of the linear pouch actuator were measured by applying a constant voltage to the pump for 1 minute and increasing the voltage by 0.5 [kV] increments until breakdown occurred (Fig.5b). No additional weights were suspended in this experiment, only a connector weighing 6.82 [g]. The bubbles generated at the onset of each breakdown were removed, and this evaluation was performed a total of five times. The data just before the fifth breakdown is plotted in Fig.S16b. The maximum stroke of 10 [mm] and pressure of 25 [kPa] were reached approximately 20 seconds after the applied voltage of 4 [kV] was applied. Fig.S16c shows the relationship between voltage and strains at each cycle. The relationship between strain ($3(L_0 - L)/(3L_0 + 4b)$) and force was also investigated (Fig.5f) In this evaluation, additional weights (50g, 100g, 200g, 300g, 400g, 500g, 1000g) were suspended from a connector weighing 6.82 [g] and 3.5 [kV] was applied to the pump.

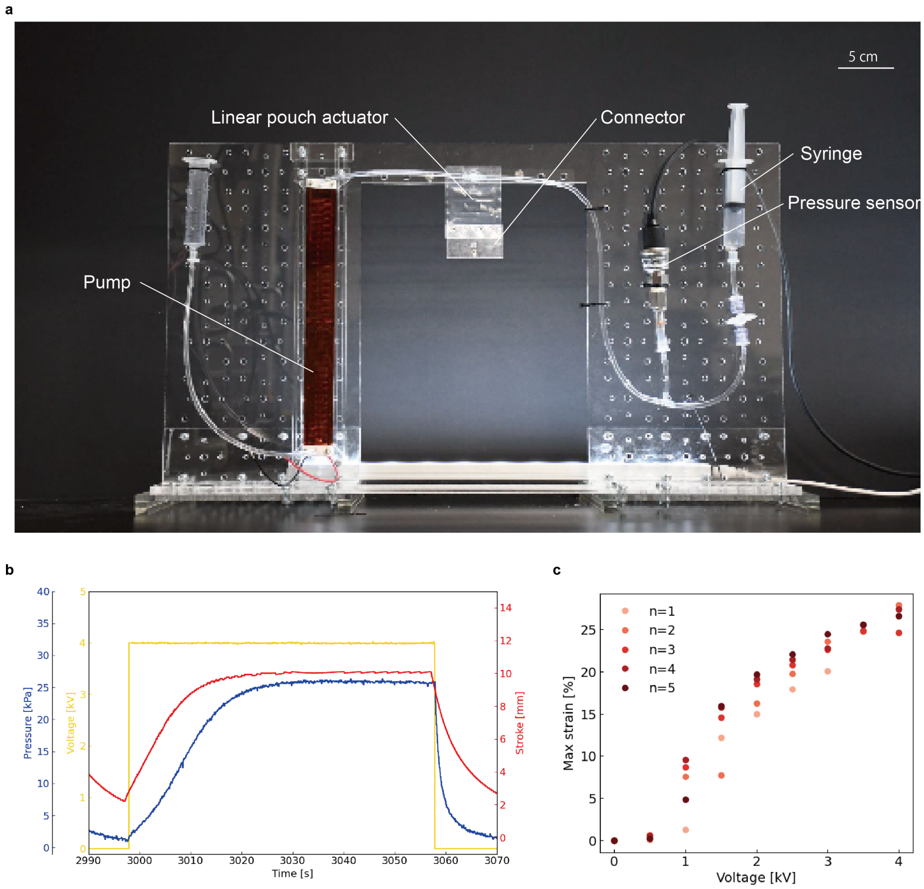

Fig. S16 Evaluation of linear pouch actuator. **a** Experimental setup. **b** Pressure and actuator's stroke response to the applied voltage. **c** Relationship between applied voltage and maximum stroke for each breakdown cycle.

Table S3: Comparison of linear pouch actuators

Reference	[7]	[8]	[9]	Our paper
Mechanism	Mechanical pump	Electrostatic force	Evaporation	EHD pump
Size of pouch actuator [cm^2]	56	24	75	12
Max strain [%]	28 (40 kPa applied)	10	36	24 (25 kPa applied)
Force at 5% strain [N]	75 (Estimated, 40 kPa applied)	1 (Estimated)	45 (Estimated)	9 (29 kPa applied)
Max strain per size [cm^{-2}]	0.50	0.42	0.48	2.00
Force per size [N cm^{-2}]	1.33	0.04	0.6	0.75

Supplementary note 6: Demonstration of McKkiben muscles-driven prosthetic hand

A prosthetic hand driven by pumps and McKibben artificial muscles was developed (Fig.S17a). Fig.S17b and c show the structure and mechanism. In this prosthetic hand, four fingers from the index to the little finger are active, and the thumb can be fixed at any angle. The finger movements are reproduced by two McKibben muscles and one pump per finger. McKibben's artificial muscle of 230 [mm] in length is placed on both sides of the pump (100 pairs, 2 mm width), and the pressure generated by the pump causes McKibben's artificial muscle on the positive pressure side to contract in the longitudinal direction. In this case, the McKibben artificial muscle on the negative pressure side acts as an actively deforming tank, so this fluid system can be configured as a closed system. In addition, by switching the polarity of the voltage to the pump, two actions, finger bending and finger opening, are possible.

This prosthetic hand can be produced relatively inexpensively because the materials and manufacturing process are not special. The link structure corresponding to the skeleton of a finger was created by inserting a shaft (parallel pin 1×12, TAIYO Stainless Spring) into a part output by a 3D printer (AGILISTA-3200, AR-M2, KEYENCE CORPORATION). The motion range of the link mechanism was limited by setting a physical limit so that the joints would not be in an aligned posture (dead point). The first joint of the finger and McKibben's artificial muscle are connected by a nylon thread (No. 5) that mimics a tendon and transmits the relaxation and contraction of McKibben's artificial muscle to the link structure. The dimensions of the joints and finger lengths were set by actually measuring the designer's hands and adjusting the values to geometrically manageable dimensions and shapes. The joints were designed with a thickness of 15 x 14 [mm] and a finger length (from base to fingertip) of 95 [mm]. McKibben's artificial muscle consists of a fibrous outer sleeve around a rubber inner tube. The McKibben is widely known as an artificial muscle that works specifically with pneumatic pressure, but it has also been reported to work with EHD pumps[3]. Applying pressure to the inner tube causes the tube diameter to expand, which in turn causes the outer sleeve to contract as the knitting angle of the outer sleeve decreases. In the McKibben muscle prosthesis used in this study, thin-film silicone tubing (inner diameter 2.5 [mm]- outer diameter 2.8 [mm]) was used for the inner tube and a PET monofilament braided with a 19° braid angle was used for the sleeve. The thin silicone tubing allows the McKibben artificial muscle to operate at low pressure. The pump and McKibben's artificial muscle are mounted on a 3 mm thick acrylic plate base in the shape of a laser-cut forearm and a forearm created by a 3D printer (PLA, raise3D).

Supplementary note 7: Demonstration of tube-formated display

Tube format display was demonstrated using our pump (10 pairs, 4mm width). The pump is equipped with a tube (LMT-55, Saint-Gobain) with an inner diameter of 2 mm and an outer diameter of 4 mm, and three terminal manual valves (model number and manufacturer) are located on both sides of the pump. After filling the system with the working liquid Novec7300 and a small amount of green-colored water, the manual valves are closed to form a completely closed system. Because of the water repellency of the working liquid Novec7300, it separates from the colored water, so the colored water can be transported indirectly by pumping the working liquid with a pump. This display works continuously by switching the polarity of the voltage and reciprocating it before the colored water reaches the electrodes. In the demonstration, the tubing and pump were attached to a cup and ±2 kV was applied to the pump.

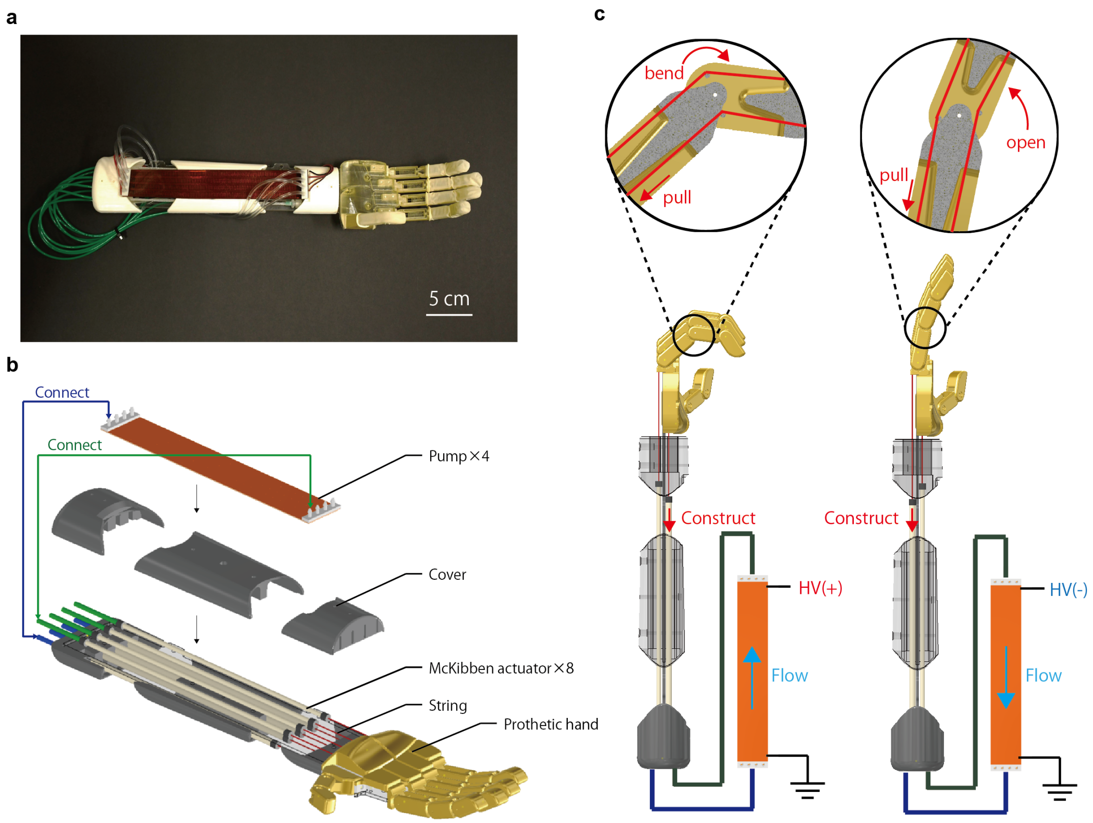

Fig. S17 System of a prosthetic hand. **a** Developed prosthetic hand. **b** The structure. **c** The driving mechainsm.

Legends for movie S1-S9

Movie S1 Visualization of EHD flow by opposing comb-shape electrodes.

In the container, new electrodes (4 pairs) are arranged and filled with Novec7300 (colorless and transparent) as the working liquid and a small amount of silicon oil (white) for flow visualization. In the first half of the movie, +4kV is applied, and in the latter half, -4kV is applied, visualizing the EHD flow in different directions.

Movie S2 Verification of passive resilience to dielectric breakdown by high voltage.

In a system in which a working liquid (Novec7300) is circulated by our pump (10 pairs, 4 mm width), the voltage applied to the pump was gradually increased. Dielectric breakdown occurred at 6 kV, and electrical discharges and bubbles were observed in the pump. After manually removing the bubbles and re-applying the voltage, the pump was able to generate flow.

Movie S3 Verification of passive resilience to dielectric breakdown by conductive liquid.

Colored water was added as an impurity to a system in which a working fluid (novec7300) was circulated by a pump (10 pairs, 4 mm width). When the colored water reaches the pump due to the flow of the pump with 3 kV applied, a dielectric breakdown occurs. We show that by removing the colored water from the pump, the pump can generate flow again.

Movie S4 Active resilient system by two pumps in series.

Normally, 3 kV is applied to the pump on the right side to generate flow. If dielectric breakdown due to air bubbles is observed, 3 kV is automatically applied to the pump on the left side to remove the bubbles from the flow path, and the system recovers. This is an example of an active resilient fluid system that assumes a dielectric breakdown that can be caused by a disturbance.

Movie S5 Jet flow by a high flow rate pump in bending condition.

A pump with 100 electrode pairs and a channel width of 8 mm was bent and fixed, with 4kV voltage applied. Despite the bent state, a jet flow was observed, demonstrating the pump's flexibility and high pumping performance.

Movie S6 Operation of a linear pouch actuator driven by a twisted pump.

A linear pouch actuator was operated by a twisted pump (100 pairs, 4 mm width). The linear actuator expanded as the working fluid flowed into the pouch by the pumps, causing linear motion. We increased the voltage applied to the pump by 0.5 kV increments until dielectric breakdown occurred. The first half of the movie shows the operation of the linear pouch actuator up to the first breakdown. In the second half, it is up to the fifth breakdown.

Movie S7 Operation of a linear pouch actuator driven by a twisted pump at loads from 50g to 1000g.

A linear pouch motor with a load attached was operated by applying 3.5 kV to a twisted pump (100 pairs, 4 mm width). The first half of the movie shows the operation with loads of 50, 100, 200, and 300 g, and the second half of the movie shows the operation with loads of 400, 500, and 1000 g.

Movie S8 Demonstration of a prosthetic hand driven by four pumps.

The developed prosthetic hand consists of 8 McKibben prosthetic muscles and 4 pumps (100 pairs, 2 mm width). This prosthetic hand can grasp and release a PET bottle (26 g) by switching the polarity of the voltage to the pump. Daily activities such as shaking hands and drinking were also demonstrated.

Movie S9 Tube-format display by transporting colored water.

This display presents information through the movement of colored water (green) inside the tube. The pump indirectly transports the colored water through the working liquid, Novec 7300, and reciprocating movement is possible by switching the polarity of the voltage. The tubing and pump (10 pairs, 4 mm width) were seamlessly attached to the cup, taking advantage of its flexibility. Furthermore, when the cup is grasped, i.e., the tube is squashed, the movement of the colored water stops, presenting information about the grasping of the object.

References

[1] M. Smith, V. Cacucciolo, H. Shea, *Science* **2023**, *379*, 6639 1327.

[2] Y. Seki, Y. Kuwajima, H. Shigemune, Y. Yamada, S. Maeda, *Journal of Robotics and Mechatronics* **2020**, *32*, 5 939.

[3] V. Cacucciolo, J. Shintake, Y. Kuwajima, S. Maeda, D. Floreano, H. Shea, *Nature* **2019**, *572*, 7770 516.

[4] W. Tang, C. Zhang, Y. Zhong, P. Zhu, Y. Hu, Z. Jiao, X. Wei, G. Lu, J. Wang, Y. Liang, et al., *Nature communications* **2021**, *12*, 1 2247.

[5] Gotec-esx04 specifications, ; available at https://www.gotec.ch/en/product/esx-04/.

[6] A. J. Veale, S. Q. Xie, I. A. Anderson, *Smart Materials and Structures* **2016**, *25*, 6 065013.

[7] R. Niiyama, X. Sun, C. Sung, B. An, D. Rus, S. Kim, *Soft Robotics* **2015**, *2*, 2 59.

[8] N. Kellaris, V. Gopaluni Venkata, G. M. Smith, S. K. Mitchell, C. Keplinger, *Science Robotics* **2018**, *3*, 14 eaar3276.

[9] K. Narumi, H. Sato, K. Nakahara, Y. ah Seong, K. Morinaga, Y. Kakehi, R. Niiyama, Y. Kawahara, *IEEE Robotics and Automation Letters* **2020**, *5*, 3 3915.
